# Phosphorylation of Mouse Immunity-Related GTPase (IRG) Resistance Proteins Is an Evasion Strategy for Virulent *Toxoplasma gondii*

**DOI:** 10.1371/journal.pbio.1000576

**Published:** 2010-12-21

**Authors:** Tobias Steinfeldt, Stephanie Könen-Waisman, Lan Tong, Nikolaus Pawlowski, Tobias Lamkemeyer, L. David Sibley, Julia P. Hunn, Jonathan C. Howard

**Affiliations:** 1 Institute for Genetics, University of Cologne, Cologne, Germany; 2 Cologne Excellence Cluster on Cellular Stress Responses in Aging-Associated Diseases (CECAD), Cologne, Germany; 3 Department of Molecular Microbiology, Washington University School of Medicine, St. Louis, Missouri, United States of America; University of Vermont, United States of America

## Abstract

Virulence of complex pathogens in mammals is generally determined by multiple components of the pathogen interacting with the functional complexity and multiple layering of the mammalian immune system. It is most unusual for the resistance of a mammalian host to be overcome by the defeat of a single defence mechanism. In this study we uncover and analyse just such a case at the molecular level, involving the widespread intracellular protozoan pathogen *Toxoplasma gondii* and one of its most important natural hosts, the house mouse (*Mus musculus*). Natural polymorphism in virulence of Eurasian *T. gondii* strains for mice has been correlated in genetic screens with the expression of polymorphic rhoptry kinases (ROP kinases) secreted into the host cell during infection. We show that the molecular targets of the virulent allelic form of ROP18 kinase are members of a family of cellular GTPases, the interferon-inducible IRG (immunity-related GTPase) proteins, known from earlier work to be essential resistance factors in mice against avirulent strains of *T. gondii*. Virulent *T. gondii* strain ROP18 kinase phosphorylates several mouse IRG proteins. We show that the parasite kinase phosphorylates host Irga6 at two threonines in the nucleotide-binding domain, biochemically inactivating the GTPase and inhibiting its accumulation and action at the *T. gondii* parasitophorous vacuole membrane. Our analysis identifies the conformationally active switch I region of the GTP-binding site as an Achilles' heel of the IRG protein pathogen-resistance mechanism. The polymorphism of ROP18 in natural *T. gondii* populations indicates the existence of a dynamic, rapidly evolving ecological relationship between parasite virulence factors and host resistance factors. This system should be unusually fruitful for analysis at both ecological and molecular levels since both *T. gondii* and the mouse are widespread and abundant in the wild and are well-established model species with excellent analytical tools available.

## Abstract 2

GTPases of the mouse IRG protein family, mediators of resistance against *Toxoplasma gondii* in the mouse, are inactivated by a polymorphic kinase of the parasite, resulting in enhanced parasite virulence.

## Author Summary

Many pathogens manipulate the immune system of their hosts to facilitate infection and ensure transmission to subsequent hosts. The intracellular protozoan *Toxoplasma gondii*, a relative of the malaria parasite, is able to infect and persist in a remarkable variety of warm-blooded hosts. Indeed roughly a third of the human race carry live *Toxoplasma* cysts in their brains with no overt effects. *Toxoplasma* infection is kept at bay in many mammals (but not in humans) by a resistance system based on a family of proteins known as the immunity-related GTPase (IRG) family. IRG proteins accumulate in infected cells on the vacuoles containing the parasite and ultimately destroy them. In this paper, we show that, in the mouse, *Toxoplasma* can oppose the IRG system by secreting an enzyme called ROP18 into infected cells, which phosphorylates key amino acids on the IRG proteins, rendering them inactive. Not all strains of *Toxoplasma* can produce an active form of ROP18, but those strains that do are more virulent. We propose that individual hosts control *Toxoplasma* with differing efficiency, and the variation we see in ROP18 kinase activity produced by different *Toxoplasma* strains is an evolutionary response to this. Thus, in different mammalian hosts, each strain seeks a balance between an excess of virulence (resulting in premature death of the host) and resistance that is too efficient (resulting in clearance of the parasite and sterile immunity).

## Introduction

*Toxoplasma gondii* is an intracellular protozoan parasite with a complex life cycle and is distantly related to the malarial genus *Plasmodium*. The sexual phase occurs only in the true cats (Felidae), while all warm-blooded animals including humans can be intermediate hosts. The infection is transmitted from cats to intermediate hosts via ingestion of oocysts from the faeces of infected cats, and from intermediate hosts back to cats by carnivory. Typically, *T. gondii* establishes a lifelong chronic infection in intermediate hosts by encysting, after an initial phase of rapid intracellular proliferation and cell–cell spread, in brain and muscle. The life cycle is completed when the infected host is eaten by a cat [1]. However, some *T. gondii* strains are highly virulent for mice, killing the host as early as ten days after initial infection. Of the three clonal lineages of *T. gondii* commonly found in Eurasia and North America [2],[3], the type I strains are highly virulent for mice [4]. In a genetic cross between a type I virulent and a type III avirulent strain, the serine-threonine kinase secreted from rhoptry organelles, ROP18 [5], emerged as a major virulence factor [6]. In another genetic cross, ROP16 kinase and the ROP5 pseudokinases were implicated in virulence differences between type II and type III strains [7]. Comparative studies of ROP18 from multiple *T. gondii* strains, including the major Eurasian types, established that this virulence protein shows extensive polymorphic sequence variation derived from recent episodes of positive selection [8].

In mice the major resistance factors preventing acute death from avirulent *T. gondii* infection, and thereby allowing *T. gondii* transmission, are large GTPases of the immunity-related GTPase (IRG) family [9],[10]. These interferon-γ (IFNγ)-inducible proteins accumulate on the parasitophorous vacuole membrane (PVM) within minutes after infection of a cell by an avirulent *T. gondii* strain [11],[12]. The PVM becomes vesiculated then disrupted, and the parasite is killed [11],[13],[14]. Encoded by about 15 active genes in the C57BL/6 mouse genome ([15] and unpublished results), the IRG proteins function interactively and nonredundantly in resistance [16], so that disruption of single members of the family cause highly significant early mortality following infection with avirulent strains [9]. However, during infection by a type I virulent *T. gondii* strain, few vacuoles accumulate IRG proteins in large amounts or disrupt [12],[14],[17],[18], and parasite replication in cells is not inhibited by IFNγ [17]. These parallel observations on parasite virulence and host resistance suggested that IRG proteins might be phosphorylated and inactivated by ROP kinases from virulent strain *T. gondii*.

The switch I and switch II loops of the GTP-binding domain of GTPases are sequence elements whose conformational changes during the GTP-binding and hydrolysis cycle largely determine the functional activity of the protein via its interaction with other proteins [19],[20]. In classical Ras-like small GTPases the switch I loop contains a conserved threonine (the “G2 threonine”) interacting with a catalytically important Mg^2+^ ion. In Irga6, the only IRG protein for which structural [21], biochemical ([22] and Pawlowski et al., unpublished data), and functional ([11] and Liesenfeld et al., unpublished data) information is available, highly conserved threonines are present in the switch I loop, but the typical Ras configuration is not seen and these residues do not coordinate the Mg^2+^ ion [21]. We show here that the ROP18 kinase from highly virulent strains targets Irga6, phosphorylating two threonines in the switch I loop. For Irga6, the switch I loop from residues 100 to 109 is implicated in the formation of a critical dimer interface with a neighbouring Irga6 molecule during the process of GTP-dependent activation and subsequent hydrolysis (Pawlowski et al., unpublished data). We present evidence that phosphorylation of either of the target threonines inactivates the GTPase and inhibits its normal accumulation on the PVM. Virulent strains also phosphorylate other IRG resistance proteins, and other ROP kinase homologues have been associated with virulence, suggesting that this will prove to be a general mechanism by which *T. gondii* moderates the IRG system, thus favouring long-term survival of both parasite and host.

## Results

**Figure 1:**
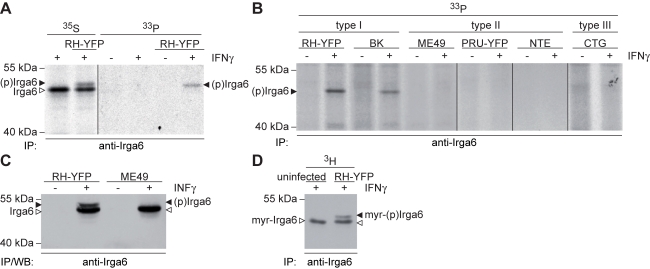
Irga6 is phosphorylated by type I virulent *T. gondii*. (A) A novel phosphorylated Irga6 band (black arrowhead) was immunoprecipitated with the monoclonal anti-Irga6 antibody, 10E7, from RH-YFP-infected, IFNγ-induced mouse L929 cells metabolically labelled with ^35^S-methionine/cysteine or ^33^P-phosphoric acid. Nonphosphorylated Irga6 is indicated with an open arrowhead. The figure is a montage from two segments of a single autoradiogram, joined as indicated by black vertical line. (B) Only virulent *T. gondii* strains phosphorylate Irga6. MEFs were induced with IFNγ, infected with virulent type I and avirulent type II and type III strains, and labelled with ^33^P-phosphoric acid. A single phosphorylated band (black arrowhead) was immunoprecipitated with 10E7 only from cells infected with type I strains. The figure is a montage of segments from several autoradiograms, joined as indicated by the black vertical lines. Phosphorylated Irga6 from cells infected with RH-YFP served as positive control for all segments (not shown). (C) The electrophoretically more slowly migrating phosphorylated band (black arrowhead), immunoprecipitated with anti-Irga6 165 antiserum from IFNγ-induced L929 cells infected with virulent strain RH-YFP, is Irga6 and not a coprecipitated protein (Western blot with 10E7). Only the faster band of Irga6 is immunoprecipitated from cells infected with avirulent ME49 strain (open arrowhead). (D) Both Irga6 bands were immunoprecipitated with 10E7 from RH-YFP-infected IFNγ-induced L929 cells labelled with ^3^H-myristic acid (autoradiogram). In the absence of infection only the lower myristoylated band is seen. Black arrowhead: myristoylated phosphorylated Irga6 (myr-(p)Irga6). Open arrowhead: myristoylated Irga6 (myr-Irga6).

We looked directly for phosphorylation of the mouse IRG protein Irga6, which we immunoprecipitated from *T. gondii*-infected fibroblasts. IFNγ-induced fibroblasts were labelled in parallel with ^33^P-phosphoric acid or ^35^S-methionine/cysteine and infected with the virulent type I RH-YFP strain *T. gondii* (Figure 1). A single phosphorylated protein was immunoprecipitated corresponding to the upper band of a doublet metabolically labelled with ^35^S-methionine/cysteine, and found only in infected cells (Figure 1A). The single phosphorylated band was found only in immunoprecipitates from virulent strain infections (Figure 1B, type I strains RH-YFP and BK). The ^33^P-labelled band is not due to another protein coprecipitated with Irga6 since it could also be detected by Western blot with monoclonal and polyclonal antibody reagents specific for Irga6 (Figure 1C and unpublished data). Irga6 is a myristoylated protein [23], and nonmyristoylated Irga6 also runs slightly slower than native myristoylated protein ([24] and unpublished data). However, both Irga6 bands in immunoprecipitates from the virulent strain infections incorporated ^3^H-myristic acid (Figure 1D), and are therefore myristoylated. Thus, infection by virulent strains of *T. gondii*, but not by avirulent strains, results in significant phosphorylation of native, myristoylated Irga6.

**Figure 2:**
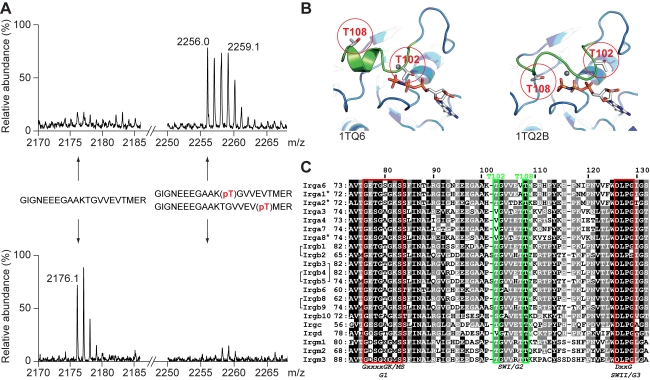
Irga6 is phosphorylated at T102 and T108 in the switch I loop. (A) MALDI-TOF spectra of tryptic digests of immunoprecipitated phosphorylated (upper profile) and nonphosphorylated (lower profile) Irga6 from IFNγ-induced, RH-infected MEFs. The partial spectra on the left are focused on the m/z 2176.1, corresponding to the nonphosphorylated tryptic Irga6 peptide G^91^IGNEEEGAAKTGVVEVTMER^111^. The partial spectra to the right are focused on m/z 2256.0, corresponding to the same peptide monophosphorylated on either T102 or T108. The isotopic pattern of m/z 2256.0 in the phosphorylated material is partially overlaid by a pattern of unknown identity at m/z 2259.1. (B) The switch I region (green) occurs in different configurations in Irga6 crystals [21] (ribbon presentation). T102 and T108 are labelled in red. In PDB structure 1TQ6, T108 is turned outwards while T102 points towards the nucleotide. In PDB 1TQ2B, T108 is turned inwards, pointing towards the γ-phosphate, while T102 is rotated away from the nucleotide (GppNHp in stick format; grey sphere: Mg^2+^ ion). (C) ClustalW alignment of the amino acid sequence between the first (G1) and the third (G3) nucleotide binding motifs (red boxes) of the mouse IRG proteins [15]. *Irga5* and *Irgb7* are both severely disabled pseudogenes and are not included in the alignment. There is to date no evidence for a protein product from *Irga1*, *Irga2*, and *Irga8* (indicated by *), while eight of the *IRGB* units are expressed in the form of four head-to-tail tandem proteins (b2-b1, b5-b4, b5^*^-b3, and b9-b8), indicated by brackets ([15] and unpublished data). Note that there are two identical copies of *Irgb5* (*Irgb5* and *Irgb5* ^*^) present in the C57BL/6 genome [15] represented by one sequence in the alignment. The switch I region is indicated below the alignment. The T108 residue of Irga6 is absolutely conserved in the other IRG proteins, while the T102 of Irga6 is largely conserved (green boxes). Amino acid positions indicated above the alignment refer to Irga6, the ones indicated on the left on the alignment refer to the respective IRG. Shading indicates degree of conservation (black: 90%, grey: 60%).

The phosphorylated sites on Irga6 were identified by mass spectrometry (MS) analysis of the phosphorylated Irga6 band immunoprecipitated from IFNγ-induced fibroblasts infected with RH-YFP. Tryptic peptides were found corresponding in mass to phosphorylated derivatives of a region of the switch I loop of the nucleotide binding domain containing two closely-spaced threonines, T102 and T108 (Figure 2). Putatively phosphorylated peptides containing both target threonines, corresponded in molecular mass exclusively to monophosphorylated derivatives (Figure 2A). To confirm the phosphorylation of the two minimal peptides, the candidates were further resolved by CID (collision-induced dissociation) MS/MS (Figure S1).

Among the crystal structures available for Irga6 [21], T102 and T108 are found in several different configurations (Figure 2B). In one structure (Protein Data Bank [PDB] 1TQ6), the side chain of T102 is close to the nucleotide, while the side chain of T108 is distant from the nucleotide and largely exposed to solvent (Figure 2B, left graphic). In another (PDB 1TQ2B), T108 is oriented towards the nucleotide, while T102 is rotated away from the nucleotide (Figure 2B, right graphic). The alternation between the two structures correlates with the presence or absence of a short helical turn in the switch I loop. Phosphorylation of one threonine may lock one configuration for the switch I loop, preventing kinase access to the second threonine. Unlike the classical G2 threonine of many small GTPases, neither T102 nor T108 interacts with the Mg^2+^ ion in any Irga6 structure so far studied [21]. T108 is absolutely conserved among all the IRG proteins, while T102 is highly but not completely conserved (Figure 2C).

**Figure 3:**
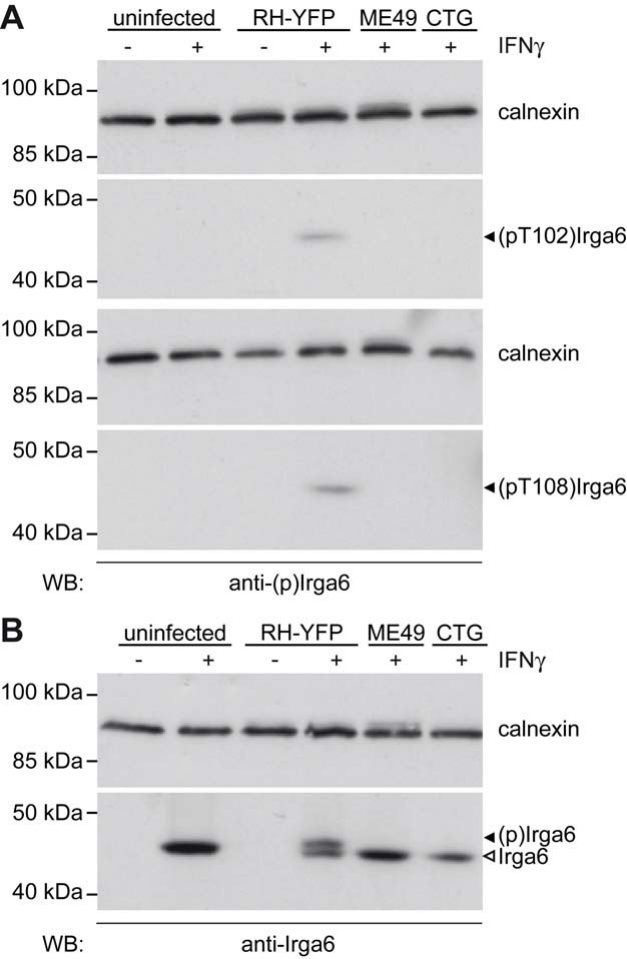
Rabbit anti-pT102 and anti-pT108 peptide antibodies are specific for phosphorylated Irga6. (A) Western blot of detergent lysates of IFNγ-induced L929 cells either uninfected or infected with type I RH-YFP, type II ME49, or type III CTG *T. gondii*. Only RH-YFP infection caused phosphorylation of Irga6, detectable as a unique band (black arrowhead) with the anti-pT102 (upper blot) or anti-pT108 antibodies (lower blot). (B) Western blot of the same lysates as in Figure 3A in which Irga6 was detected with the monoclonal anti-Irga6 antibody 10E7. The phosphorylated slower-migrating band is detectable only in the lysate from cells infected with virulent RH-YFP. Black arrowhead: phosphorylated Irga6 ((p)Irga6). Open arrowhead: Irga6. Note that the detectability of the phosphorylated bands by the anti-pT102 and anti-pT108 antibodies in (A) is independent of variations in the level of total Irga6.

That T102 and T108 are targets of phosphorylation by virulent *T. gondii* strains in infected cells was confirmed by Western blot of *T. gondii*-infected, IFNγ-induced cells with two rabbit antisera raised respectively against peptides of Irga6 containing phospho-T102 and phospho-T108. After affinity purification both antibodies identified a single band in IFNγ-induced wild-type (wt) fibroblasts infected with virulent RH-YFP strain *T. gondii*. This band was not found in uninduced, infected fibroblasts nor in induced, uninfected fibroblasts. Most significantly, it was also not found in IFNγ-induced fibroblasts infected with the avirulent strains ME49 (type II) and CTG (type III) (Figure 3A). The same cell lysates were probed with an anti-Irga6 antibody to confirm the expression of Irga6 in the IFNγ-induced samples (Figure 3B). The specificity of the two anti-phosphopeptide reagents for phosphorylated Irga6 was further confirmed by Western blot on IFNγ-induced *Irga6*-deficient fibroblasts infected with RH-YFP strain parasites, where no signals were detected (2A).

**Figure 4:**
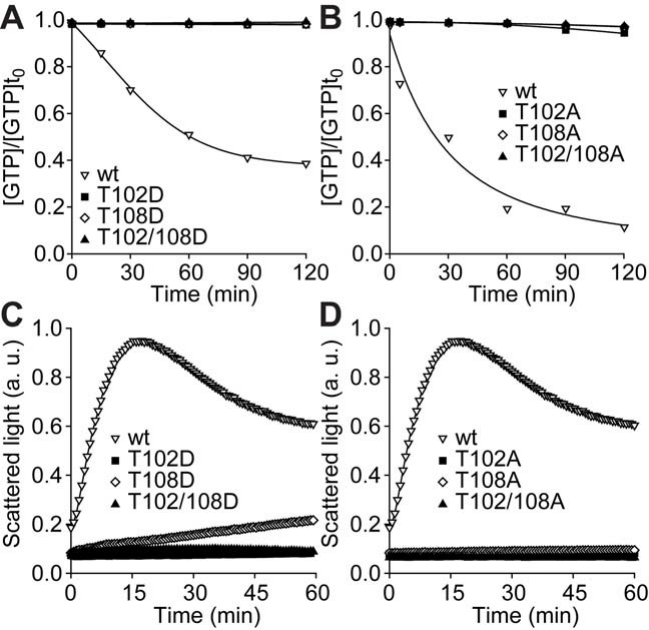
Phosphomimetic and neutral mutations of T102 and T108 disable Irga6 biochemically. (A and B) GTP hydrolysis by recombinant Irga6 is lost by phosphomimetic Thr to Asp (A) and neutral Thr to Ala (B) mutations. (C and D) GTP-dependent oligomerisation of recombinant Irga6 is lost by Thr to Asp (C) and Thr to Ala (D) mutations. Very weak oligomerisation is retained by T108D.

To examine the probable impact of phosphorylation at the two target threonines we assayed the functional properties of two sets of bacterially expressed mutant Irga6 proteins: (1) negatively charged, phosphomimetic aspartic acid mutants T102D, T108D and the double mutant T102/108D and (2) neutral alanine mutants T102A, T108A and T102/108A, compared with the wt Irga6 protein [22]. All the T102 and T108 mutations, whether phosphomimetic or neutral, essentially abolished GTP hydrolysis (Figure 4A and 4B) and strongly inhibited GTP-dependent oligomerisation (Figure 4C and 4D). The affinities of all the Thr to Ala mutants were in the wt range for both GTP and GDP, while the affinities of the T102D and T102/108D mutants for GDP were about an order of magnitude lower than wt (Table S1).

**Figure 5:**
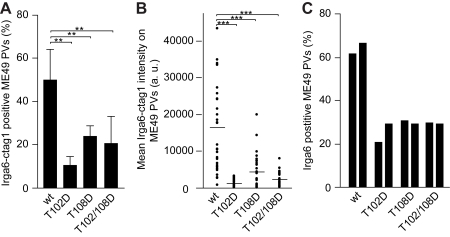
Phosphomimetic Irga6 mutants load onto the PVM inefficiently and inhibit loading of wt Irga6. Wt and phosphomimetic Irga6 mutants T102D and T108D, all tagged with ctag1, were transfected into IFNγ-induced C57BL/6 MEFs. Cells were infected 24 h later with *T. gondii* strain ME49 for 2 h and vacuoles assayed for loading of the transfected proteins (A and B) or total Irga6 (C). (A) Frequency of vacuoles loaded with transfected wt or phosphomimetic mutant Irga6 proteins (mean and standard deviation of four independent experiments, 100 vacuoles counted per experiment). (B) Loading intensity of transfected wt or phosphomimetic mutant Irga6 proteins (40 vacuoles analysed; *** *p*<0.0001; horizontal lines indicate means). (C) Frequency of vacuoles loaded with total Irga6 detected with the monoclonal anti-Irga6 antibody 10D7. The transfected phosphomimetic mutant proteins reduced the frequency of vacuoles loaded with endogenous Irga6 (results of two independent experiments, 100 vacuoles counted per experiment).

For further functional analysis, we expressed the six mutant proteins and wt Irga6, tagged at the C terminus, by transfection in IFNγ-induced mouse embryonic fibroblasts (MEFs) infected with the avirulent ME49 strain *T. gondii*. The proportion of vacuoles detectably loaded with the mutant Irga6 proteins was in all cases lower than the values for the tagged wt Irga6 (shown for the phosphomimetic Thr to Asp mutants, Figure 5A), though in no case eliminated. The amount of the mutant Irga6 proteins accumulated onto individual loaded vacuoles was, however, greatly reduced (Figure 5B). In addition, the mutant proteins had a weak but consistent dominant negative action on the accumulation of wt Irga6 on the PVM, perhaps by interference with homomeric or heteromeric oligomerisation of active Irga6 protein at the PVM (Figure 5C). This could contribute to the biological effect of the kinase even if not all target IRG molecules are phosphorylated. In conclusion, Irga6 mutated at T102 or T108 is inactive biochemically, is impaired in access to the PVM compared with wt protein, and inhibits to some degree the loading of wt Irga6 onto the PVM. The fact that the Thr to Ala mutants were essentially as impaired as the phosphomimetic Thr to Asp mutants (Figures 4 and 5; unpublished data) indicates that phosphorylation acts by interfering with the function of two essential threonines in the switch I region of Irga6, rather than through some active property conferred by the additional negative charge or of the phosphate group itself.

**Figure 6:**
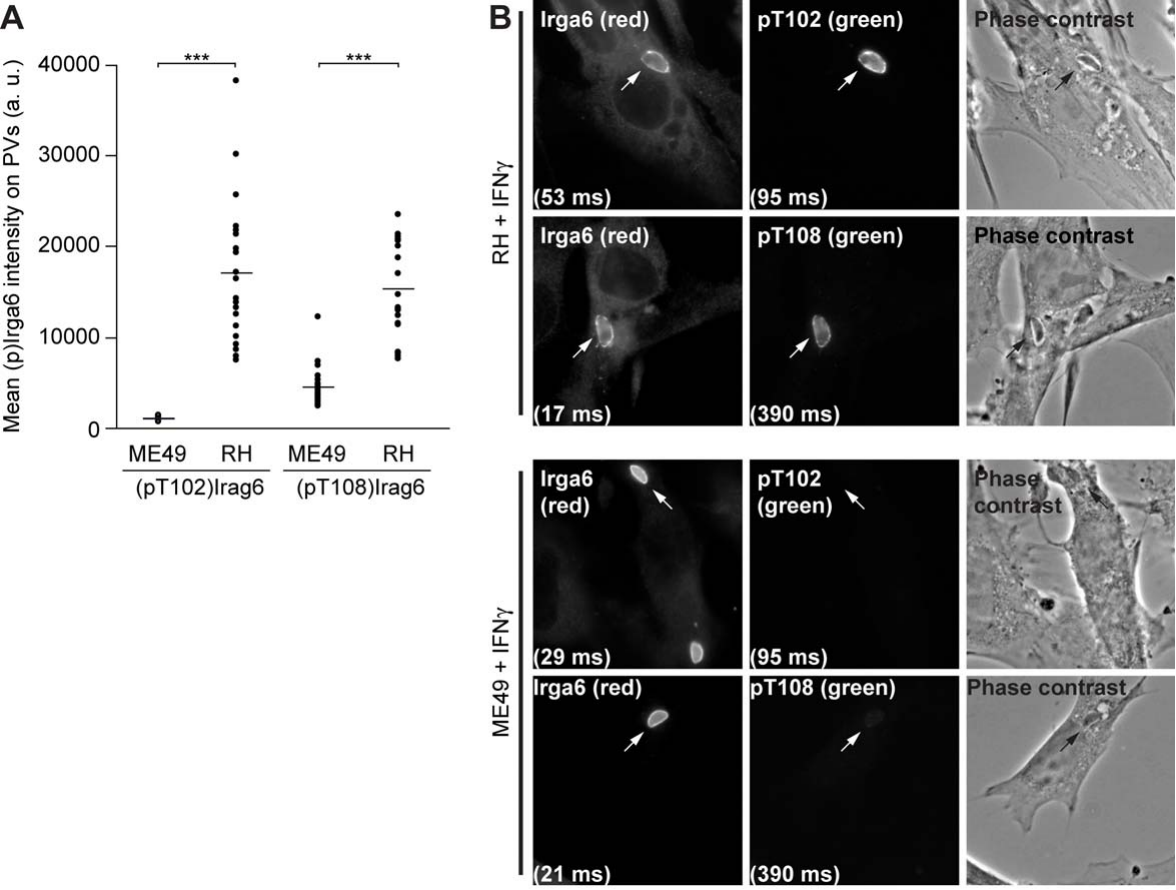
Phosphorylated Irga6 is detected at the PVM in cells infected with virulent *T. gondii*. IFNγ-induced MEFs were infected with the indicated *T. gondii* strains for 2 h and immunostained with the anti-pT102 and anti-pT108 antibodies. (A) Staining intensities at virulent RH and avirulent ME49 strain vacuoles by anti-pT102 and anti-pT108 antibodies. (*** *p*<0.0001; horizontal lines indicate means). Weak but significant staining of ME49 vacuoles was recorded from the pT108-specific antibody. One representative out of three independent experiments is shown. (B) Representative immunofluorescence images of total active Irga6 (first column, 10D7) and phosphorylated Irga6 (second column, anti-pT102 and anti-pT108 antibodies) on RH (upper two rows) and ME49 (lower two rows) strain parasitophorous vacuoles. Intracellular *T. gondii* are indicated by arrows and individual exposure times are indicated in brackets. Corresponding phase contrast images are also given (third column).

The anti-pT102 and anti-pT108 antibodies detected phosphorylated Irga6 by immunofluorescence on the PVM of virulent RH strain *T. gondii* in IFNγ-induced cells. Irga6 normally accumulates on the majority of virulent strain vacuoles, but the amount of Irga6 accumulated is much lower than on the vacuoles of avirulent strains [12]. Both anti-phosphopeptide antibodies stained the majority of RH vacuoles (Figure 6A and 6B). Significant but much weaker staining of Irga6(pT108), but not Irga6(pT102), was also present on some vacuoles of the avirulent ME49 strain: the ME49 vacuole stained with anti-pT108 in Figure 6B (bottom right image) is a barely visible example. The basis for the weak but significant signals of the anti-pT108 antibodies on ME49 vacuoles is not yet clear. No staining of either anti-pT102 or anti-pT108 antibodies was seen on virulent strain vacuoles in IFNγ-induced cells from *Irga6*-deficient mice (Figure S2B) or on virulent or avirulent strain vacuoles in uninduced cells (Figure S3).

**Figure 7:**
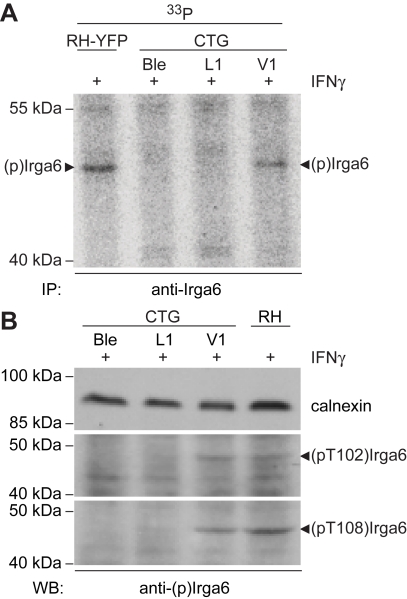
Avirulent CTG strain transgenic for virulent ROP18 phosphorylates Irga6 at T102 and T108. (A) Irga6 immunoprecipitated from ^33^P-phosphoric acid–labelled, IFNγ-induced L929 cells infected with CTG strain transgenic for empty vector (CTG Ble), kinase-dead ROP18-D394A (CTG L1) or active ROP18 from GT-1 strain (CTG V1) (autoradiogram). Only CTG V1 can phosphorylate Irga6 (black arrowhead). Virulent RH-YFP is a positive control. (B) Detergent lysates from IFNγ-induced L929 cells infected with CTG Ble, CTG L1, CTG V1, or RH-YFP were stained in a Western blot with rabbit anti-pT108 or anti-T102 antibodies. Only CTG V1 and positive control RH-YFP infected cells show the typical band of phosphorylated Irga6 (black arrowhead).

**Figure 8:**
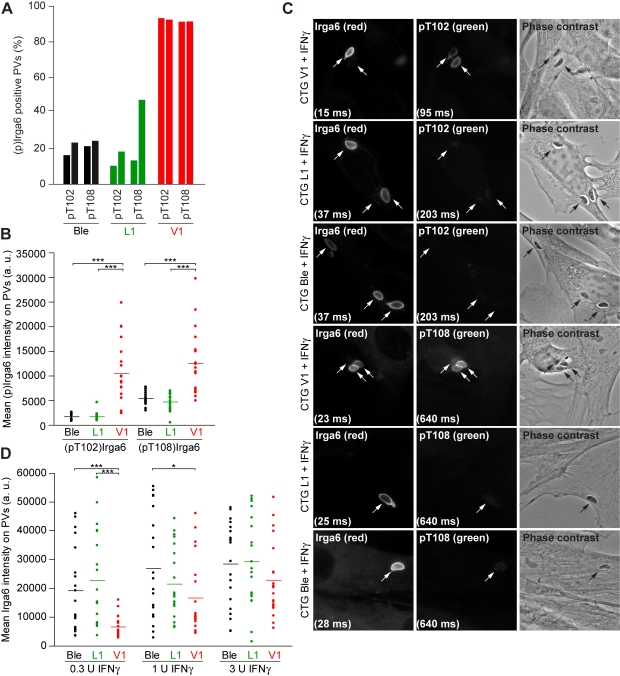
Avirulent CTG strain transgenic for virulent ROP18 phosphorylates Irga6 at the PVM. (A) Frequency of vacuoles positive for phosphorylated Irga6 detected by immunofluorescence with anti-pT102 and anti-pT108 antibodies in IFNγ-induced MEFs infected with CTG Ble (black), CTG L1 (green), or CTG V1 (red) transgenic *T. gondii* strains (results of two independent experiments, 100 vacuoles counted per experiment). (B) Intensity of phosphorylated Ira6 detected by immunofluorescence with anti-pT102 and anti-pT108 antibodies in IFNγ-induced MEFs infected with CTG Ble (black), CTG L1 (green), or CTG V1 (red) transgenic *T. gondii* strains (20 vacuoles counted; horizontal lines indicate mean signal intensities; *** *p*<0.0001). (C) Immunofluorescent images of total active Irga6 (first column, detected with monoclonal anti-Irga6 antibody 10D7) and phosphorylated Irga6 (second column, detected with anti-pT102 and anti-pT108 antibodies) on CTG V1, Ble, and L1 vacuoles in IFNγ-induced MEFs. Intracellular *T. gondii* are indicated by arrows and individual exposure times are indicated in brackets. Corresponding phase contrast images are given (third column). (D) Intensity of Irga6 loaded onto parasitophorous vacuoles in IFNγ-induced MEFs infected with CTG Ble (black), CTG L1 (green), or CTG V1 (red) transgenic *T. gondii* strains, detected with monoclonal anti-Irga6 antibody 10D7. Loading of Irga6 is inhibited by CTG V1; the effect is greatest at low IFNγ concentrations (0.3 U/ml) (horizontal lines indicate mean signal intensities; *** *p*<0.0001, * *p*<0.025). One representative out of two independent experiments is shown.

ROP18 is an active *T. gondii* kinase that occurs in different allelic forms in the three Eurasian and North American clonal lineages [8]. The protein is secreted directly from apical secretory organelles (rhoptries) into the cytosol locally at the site of cell invasion, and it accumulates on the cytosolic face of the PVM of the invading organism [5],[25]. It is thus well-placed to interfere with the accumulation of IRG proteins on the PVM. If ROP18 is responsible for the phosphorylation of Irga6 in vivo, this property should be conferred on a type III avirulent strain (CTG) transgenic for expression of active ROP18 from the virulent type I strain, GT-1, which is identical to RH strain ROP18 in sequence ([5] and TGGT1_063760 on ToxoDB, http://toxodb.org). Indeed this strain has been shown to gain virulence both in vivo [6] and in vitro [26]. We therefore infected IFNγ-induced primary MEFs with the CTG-ROP18 transgenic (CTG V1), and, as controls, with CTG transgenic for a kinase-dead mutant of ROP18 (ROP18-D394A; CTG L1) and CTG transgenic for the empty vector (CTG Ble) [6]. We detected phosphorylation of Irga6 by metabolic labelling with inorganic ^33^P-phosphate and autoradiography only from cells infected with the CTG strain transgenic for the active kinase (CTG V1, Figure 7A), and the phosphothreonine-specific antibodies also detected a signal in Western blot only in lysates from the CTG V1 strain–infected cells (Figure 7B). Strong immunofluorescent staining by anti-pT102 and anti-pT108 antibodies was seen on vacuoles from the active ROP18 transgenic (Figure 8). The great majority of CTG V1 vacuoles were stained (Figure 8A, red), while few vacuoles of the kinase-dead ROP18 (CTG L1, green) and vector only (CTG Ble, black) transgenic *T. gondii* strains were stained. Furthermore, the intensity of staining of the phosphothreonine antibodies was also strikingly higher on the CTG V1 vacuoles (Figure 8B and 8C). No staining of the anti-phosphothreonine antibodies was detected on any of the transgenic CTG strain vacuoles in cells not induced with IFNγ (Figure S4). However, significant but weak staining with anti-pT108 antibody was seen on the vacuoles of the CTG strains transgenic for the empty vector and for the kinase-dead ROP18 in IFNγ-induced cells (Figure 8B), echoing the weak staining of ME49 vacuoles by anti-pT108 antibody already noted in Figure 6. That phosphorylation of Irga6 by virulent ROP18 interferes with Irga6 function, already hinted at by the weak dominant negative effect of the Thr to Asp phosphomimetic mutant proteins shown in Figure 5C, is reflected in a drop in the overall intensity of Irga6 on the vacuoles of cells infected with the CTG V1 strain relative to the staining seen with the two control strains especially at low IFNγ concentrations (Figure 8D). We consider it likely that phosphorylation of Irga6 by virulent ROP18 occurs at the vacuole, and that the reduced loading observed is due to destabilization of interactions between IRG molecules and possibly also to conformational changes affecting the positioning of the myristoyl group required for vacuolar association of Irga6 ([24] and unpublished data).

**Figure 9:**
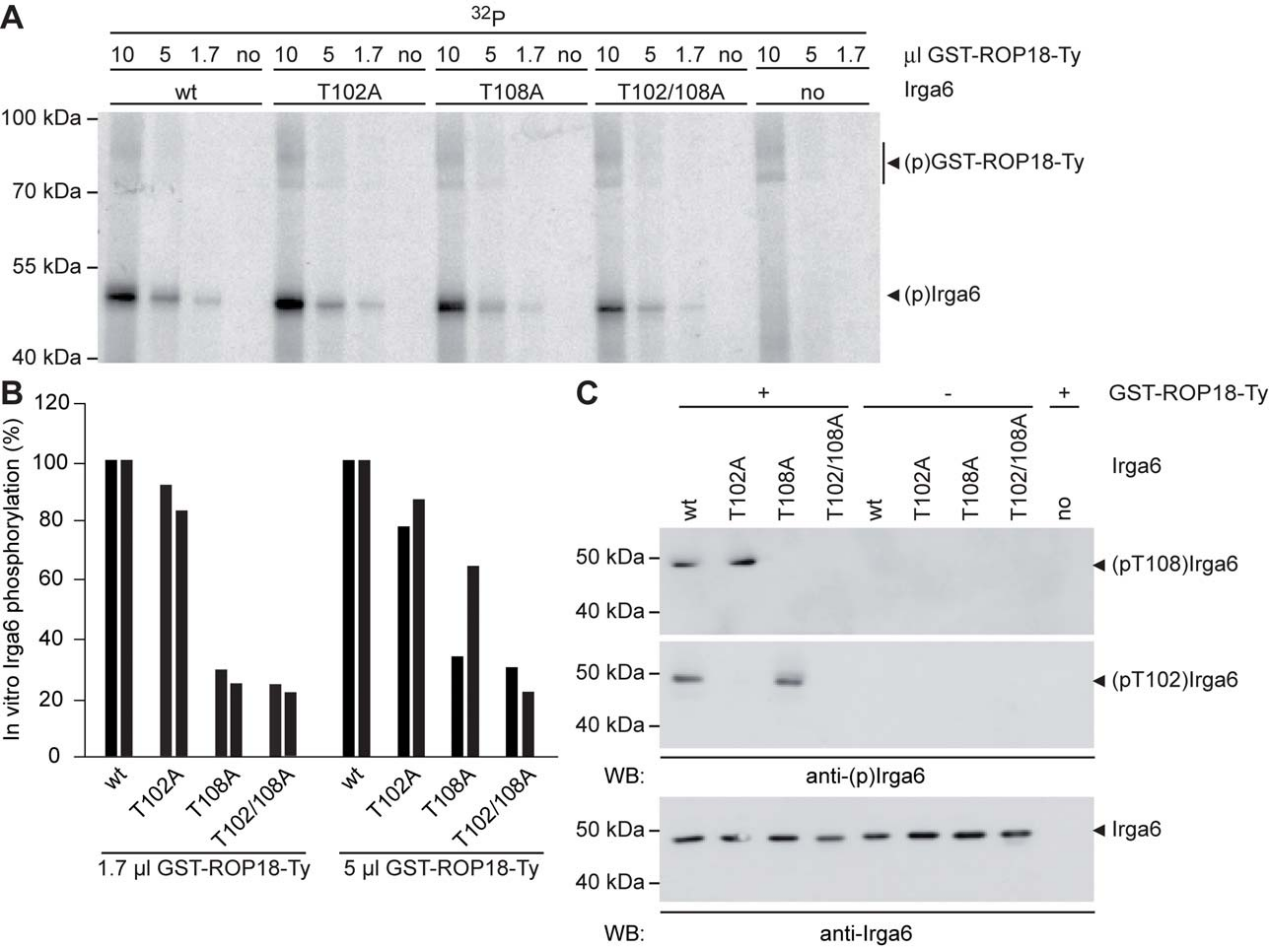
ROP18 directly phosphorylates Irga6 at T102 and T108 in vitro. (A) Bacterially expressed, purified wt or T102A and T108A mutant Irga6 proteins (300 ng) were coincubated with bacterially expressed, purified GST-ROP18-Ty (10 µl, 5 µl, 1.7 µl respectively; 1 µl ∼20 ng) in the presence of γ^32^P-ATP in vitro. The autoradiogram reveals efficient phosphorylation of wt Irga6 and a decrease in signal intensity for all Irga6 alanine mutants. This effect is most apparent at low ROP18 concentrations (1.7 µl). Autophosphorylation of ROP18 ((p)ROP18) is visible at higher kinase concentrations (10 µl and unpublished data) by the appearance of two bands (as previously observed for immunoprecipitated endogenous ROP18 [55]). (B) Quantification of signal intensities observed in (A) for two different ROP18 concentrations (5 µl and 1.7 µl) by phosphorimaging. Black bars represent two independent experiments. (C) Bacterially expressed purified wt or T102A and T108A mutant Irga6 proteins (300 ng) were coincubated with GST-ROP18-Ty (10 µl) in the presence of 1 mM unlabelled ATP in vitro. One-third of each kinase reaction was resolved by SDS-PAGE and subjected to Western blot analysis using the anti-pT102 and anti-pT108 antibodies (upper two blots) or a monoclonal anti-Irga6 antibody, 10E7 (lower blot). Alanine mutation of the target threonines in Irga6 (T102 and T108) specifically blocks recognition by the corresponding antibody.

The data presented above showed that the presence of virulent strain ROP18 is required for in vivo phosphorylation of T102 and T108 of Irga6, but did not show a direct interaction between ROP18 and Irga6. A direct interaction could, however, be shown in a yeast two-hybrid assay between both wt and kinase-dead ROP18 (Figure S5). To demonstrate that type I ROP18 is in fact an Irga6 kinase with the appropriate specificity, we coincubated bacterially derived purified full-length mature GST-ROP18-Ty fusion protein with bacterially derived purified wt Irga6 protein and the T102A, T108A, and T102/108A mutant proteins in an in vitro kinase assay, followed by autoradiography on the products (Figure 9A). The wt and all three mutant proteins were phosphorylated by ROP18. However, quantitation of the signal showed a small but consistent reduction for the T102A mutant and highly significant reductions for both the T108A and the double mutant (Figure 9B). Little ROP18 autophosphorylation was detected at the low ROP18 input concentrations used (indicated in Figure 9A). Confirmation that both T102 and T108 are direct targets for phosphorylation by type I ROP18 was provided by strong signals in a Western blot for the anti-pT102 and anti-pT108 antibodies on Irga6 from a parallel in vitro kinase assay performed with a nonradioactive source of phosphate (Figure 9C). No signals were detected in this assay by each reagent on its specific mutant protein, and from neither reagent on the double mutant protein T102/108A, confirming the specificity of the assay system. We conclude that type I ROP18 is indeed a specific and direct kinase of Irga6 T102 and T108. In vitro, ROP18 clearly also has another minor target of phosphorylation on Irga6 that we have not identified.

The IRG system specifies a number of proteins that act cooperatively in organising the destruction of the *T. gondii* vacuole. It is therefore of interest that a parallel study has identified a second IRG protein, Irgb6, as a target for virulent ROP18 kinase, with strong indications that threonines of the switch I loop are the targets [27]. In our own studies we can confirm that Irgb6 is also phosphorylated in IFNγ-induced cells infected by the virulent RH-YFP strain, although the level of phosphorylation is considerably lower than that of Irga6 (Figure S6A). Likewise, Irgb10 is phosphorylated by RH-YFP, but in IFNγ-induced cells though very weakly above a significant level of intrinsic, infection-independent phosphorylation (Figure S6B). Strong phosphorylation of Irgb10 dependent on RH-YFP infection was, however, seen in cells transfected with a plasmid expressing Irgb10 (Figure S6C). Some infection-independent phosphorylation of Irgb10 was also apparent in this experiment. Further analysis is required to establish whether the phosphorylation of Irgb10 involves the same target residues as phosphorylation of Irga6 by virulent ROP18.

## Discussion

Taken together, our results strongly support the hypothesis that the virulence of type I *T. gondii* strains for mice is due at least in part to their ability to inactivate host IRG resistance proteins by targeted phosphorylation of threonine residues in the switch I loop by ROP18 kinase. Threonine residues form part of the catalytic interface essential for the formation of the GTP-dependent active dimer, and their modification, like that of all the residues in the catalytic interface (Pawlowski et al., unpublished data), would be expected to inactivate the resistance protein. The threonine corresponding to T108 is conserved throughout the entire family of IRG proteins in the mouse, and in Irga6 appears to be quantitatively the major target of phosphorylation by type I ROP18 (Figure 9). We and Fentress et al. [27] have shown both Irgb6 and Irgb10 can be phosphorylated and Fentress et al. have shown that Irgb6 can be phosphorylated by ROP18 in the switch I loop, predominantly at the threonine homologous to Irga6 T102. Polymorphism in ROP18 kinase is evidently not the only factor responsible for reduced IRG protein loading onto the PVM of virulent strains since the effect is robust to IFNγ concentrations over 100 U/ml with native virulent strains (Figure 6 and [12],[17]) but detectable only at IFNγ concentrations below 3 U/ml with the avirulent CTG strain transgenic for ROP18 from a virulent strain (Figure 8D).

In earlier studies, we [12],[17] and another group [18] showed that the loading of IRG proteins onto the virulent *T. gondii* vacuole was seriously impaired, in vitro in IFNγ-induced fibroblasts, and in vivo in primed peritoneal macrophages. Irga6 loading intensity was substantially reduced, though the number of Irga6-positive vacuoles was not much affected, while the loading of Irgb6 and Irgb10 was completely eliminated on the great majority of vacuoles. At that time, both groups tried to show that virulent-strain ROP18 kinase was responsible for these effects, we by transfection of mature virulent ROP18 from RH strain into cells infected with the avirulent type II strain, ME49 [12], the other group by infection in vitro with the same avirulent type III CTG strain transgenic for virulent GT-1 strain ROP18, and the two control strains used in the present report [18]. Neither group saw any diminution in IRG protein loading and consequently concluded that ROP18 was not responsible for the differential loading of IRG proteins. How are these earlier findings to be reconciled with the present data, and with the data of Fentress et al. [27]? Our present view is that the earlier failures to show an effect of virulent ROP18 were due to the very high level of cytokine-induced activation of the target cells, in our case with 200 U IFNγ/ml, in the case of the other group following an intense in vivo priming regimen [18]. The present experiments show a highly significant effect of the CTG virulent ROP18 transgenic strain (CTG V1) on Irga6 loading only at an IFNγ dose of 0.3 U/ml (Figure 8D). Thus virulent ROP18 alone is apparently unable to recapitulate the striking failures of IRG protein loading onto the vacuoles seen even at very high levels of cytokine activation during infection with native virulent type I *T. gondii* strains. Nevertheless, the transgenic virulent ROP18 has been shown to have a substantial influence on virulence in a mouse mortality assay [6] and on growth and survival of the parasites in IFNγ-induced macrophages [27]. A plausible conclusion is that there are other factors in *T. gondii*, over and above ROP18, that can interfere with IRG protein loading onto the vacuoles of type I virulent strains. Future experiments should be directed towards the identification of these factors.

For some time now, it has been clear that the striking virulence differential for mice between the three clonal lineages of *T. gondii* abundant in Eurasia and North America is not reflected in anything comparable in terms of their virulence for humans. There are important clinical differences but it has not been possible to designate these clearly as differences in virulence [26]. Our findings offer an interesting form of explanation for this well-established observation since the molecular targets of ROP18 in mouse cells, IFN-inducible IRG proteins, are not present in humans, having been lost in the primate lineage before the separation of the Old and New World monkeys [15],[28]. Humans organise their resistance to *T. gondii* along other lines, perhaps involving IFNγ-induced depletion of the essential amino acid tryptophan [29],[30], for which *T. gondii* is auxotrophic, as well as other mechanisms [31],[32]. The p65 guanylate binding proteins (GBPs) are also plausible candidates for a human resistance mechanism against *T. gondii* [33]. However, these proteins are not homologous to the IRG proteins and a role in *T. gondii* resistance remains to be shown. It is possible therefore that the ROP18 kinase has coevolved with the IRG resistance system, so that in humans, in the absence of the IRG system, the ROP18 kinase is nonfunctional and its polymorphism irrelevant. It should be noted that, although the p65 GBPs have a G2 threonine residue in the switch I loop [34], there is no significant homology with IRG proteins elsewhere in the switch I loop [35] or indeed elsewhere in the entire protein that would suggest they would be targeted by a common kinase.

The ROP18 alleles of the three clonal lineages differ strikingly from each other, and there is internal evidence that the protein has been under recent positive selection [8]. The type II allelic product differs from the type I product by 22 amino acids out of 541. Type II ROP18 RNA is expressed at essentially the same level as the type I ROP18 RNA [8] but our experiments show that type II strain ROP18 protein is apparently relatively incompetent to phosphorylate either Irga6 or Irgb6 in IFNγ-induced cells. The type II ROP18 is, however, an active kinase (T. Steinfeldt, unpublished results), and weak but detectable phosphorylation of Irga6 was detected in IFNγ-induced cells infected with avirulent type II and III strains. It is not yet clear whether this low level of phosphorylation is due to type II or type III ROP18 kinase, respectively. The type III allelic product differs from the type I product at 78 positions, but perhaps more important, its expression level is very greatly reduced by a promoter modification and as a consequence it is considered a null allele [6],[8] though it is not known whether the encoded protein has kinase activity. If the ROP18 system has been under recent positive selection, it is likely that small rodents have played an important role as intermediate hosts because of the recent overwhelming dominance of the domestic cat in determining the rate of *T. gondii* evolution. The IRG system is well developed in this taxonomic group, plausibly in response to selection pressure from *T. gondii*. Thus a functional explanation for the polymorphism in the ROP18 virulence system may be found in a better understanding of the specificity and mode of action of IRG proteins. Recent (unpublished) studies from our laboratory have shown that the IRG system of mice is itself highly polymorphic both structurally and functionally, as is susceptibility to virulent strain *T. gondii* infection. The fact that ROP18 is polymorphic may indicate that different alleles are optimised for different targets, perhaps for other mouse IRG protein sequences or IRG proteins from other evolutionarily significant intermediate host species. These considerations also suggest an explanation for the existence of highly virulent *T. gondii* strains in the wild, where they may have a selective advantage in confrontations with mice carrying highly resistant *IRG* alleles capable of providing sterile immunity against less virulent strains.

*T. gondii* has extensively diversified the ROP kinase family since its separation from its distant apicomplexan cousin, *Plasmodium*, in which these proteins are not found. Other members of the family, some of which have been described as pseudokinases because of destructive modification of the catalytic site, have also been implicated in virulence by genetic studies [7]. Polymorphic ROP16 kinase has recently been shown to act as a tyrosine kinase affecting the host cell inflammatory pathways regulated by STAT3 [36] and STAT6 [37], probably by direct phosphorylation and activation of these latent transcription factors. Future studies will reveal whether ROP18 is the only member of the ROP kinase family dedicated to the control of the genetically complex and rapidly evolving IRG resistance system, not only in the mouse but also in most mammalian groups outside the higher primates [15],[28]. The IRG resistance system may be modulated to a greater or lesser extent by ROP kinases and pseudokinases of all *T. gondii* strains, each seeking in different mammalian hosts a balance between an excess of virulence, resulting in premature death of the host, and too efficient resistance, resulting in clearance of the parasite and sterile immunity.

The inactivation of IRG proteins by phosphorylation of essential switch I threonines appears to be a potential Achilles heel for resistance mediated by IFNγ-inducible GTPases. The switch I and II loops of Rho family GTPases have been extensively targeted by bacterial glucosylating, deamidating, and ADP-ribosylating enzymes to favour or inhibit phagocytic uptake [38]. However, the destructive phosphorylation of switch I threonines of IRG proteins mediated by *T. gondii* ROP18 kinase appears to be a novel virulence mechanism, in this case targeted against a dedicated resistance system rather than a housekeeping protein, and in this sense resembling in principle the “gene-for-gene” virulence-resistance systems [39] characteristic of R-gene–mediated immunity in plants [40].

## Materials and Methods

### *T. gondii* Strains, In Vitro Passage and Infection of Murine Fibroblasts

Tachyzoites from *T. gondii* type I virulent strains RH-YFP [41] and RH [42], BK [43], type II avirulent strains ME49 [44], NTE [45] and PRU-YFP [41], and type III avirulent strain CTG [46] were maintained by serial passage in confluent monolayers of HS27 cells [11]. Tachyzoites from *T. gondii* transgenic strains CTG Ble (a type III strain containing a Ble^R^ selectable marker as a control), CTG L1 (CTG expressing a kinase-dead mutant form (ROP18-D394A) of the type I GT-1 allele of ROP18 [5], and CTG V1 (CTG expressing an active form of the type I GT-1 allele of ROP18) [6] were passaged in HS27 cells as described for the other *T. gondii* strains. All tachyzoites were used immediately after harvest for inoculation of untreated, IFNγ-stimulated, and/or transiently transfected fibroblasts at a multiplicity of infection of 5 to 10 [11].

### Cell Culture

Wt and *Irga6*-deficient [11] MEFs prepared from 14-d wt C57BL/6 embryos were maintained in DMEM, high glucose (Invitrogen), supplemented as described above but with 10% FCS (Biochrom). L929 mouse fibroblasts were cultured in IMDM (Invitrogen) containing 1× MEM nonessential amino acids, 100 U/ml penicillin, 100 µg/ml streptomycin supplemented with 10% FCS as previously described [14]. Cells were induced for 24 h with 200 U/ml IFNγ (Cell Concepts) unless indicated otherwise.

### Cell Extracts and Western Blot Analysis

1–2×10^5^ MEFs or 2–4×10^5^ L929 cells were seeded to individual wells of a six-well plate, induced with IFNγ or transiently transfected. Cells were harvested by scraping and centrifugation and were lysed in 100 µl per well of 0.5% NP-40, 150 mM NaCl, 20 mM Tris/HCl (pH 7.6), 2 mM MgCl_2_ supplemented with protease and phosphatase inhibitors (Complete, Mini, EDTA free and PhosSTOP, Roche) for 30 min on ice. Postnuclear lysates were subjected to SDS-PAGE and Western blot.

### Expression Constructs

Wt and mutant Irga6 proteins were expressed from pGW1H and pGEX-4T-2 vectors in mouse fibroblasts and *Escherichia coli* BL21 respectively. The wt constructs were pGW1H-Irga6-ctag1 [16],[24] and pGEX-4T-2-Irga6 [22], respectively, and the desired mutants were generated in these constructs by site-directed mutagenesis using the Quick-Change protocol (Stratagene). The following mutant Irga6 proteins were generated: Irga6-T102A, -T108A, -T102/108A, -T102D, -T108D and -T102/108D. Ctag1 was used as C-terminal epitope tag [24].

For expression in mouse fibroblasts, pGW1H constructs were transfected using FuGENE6 (Roche) according to the manufacturer's instructions. For biochemical analysis, proteins were expressed from pGEX-4T-2 constructs as N-terminal GST fusion protein and purified as described earlier [16]. The C-terminally Ty-tagged mature form of RH-derived ROP18 was cloned via SalI restriction from pGW1H-ROP18-Ty RH-YFP [12] into the bacterial and yeast expression vectors pGEX-4T-2 (GE Healthcare) and pGAD-C3/pGBD-C3 [47], respectively. Generation of pGAD-/pGBD-Irga6 was described previously [16]. The kinase-dead ROP18-D394A mutant (numbering refers to unprocessed form) was generated by site-directed mutagenesis. Primers used were (including reverse complement sequences): Irga6: T102A: 5′-gggaatgaagaagaaggtgcagctaaagctggggtggtggaggtaaccatgg-3′; T102D: 5′-gggaatgaagaagaaggtgcagctaaagatggggtggtggaggtaaccatgg-3′; T108A: 5′- gctaaaactggggtggtggaggtagccatggaaagacatccatac-3′; T108D: 5′-gctaaaactggggtggtggaggtagacatggaaagacatccatac-3′; T102/108A: 5′-gaatgaagaagaaggtgcagctaaagctggggtggtggaggtagccatggaaagacatccatacaaacac-3′; T102/108D: 5′-gaatgaagaagaaggtgcagctaaagatggggtggtggaggtagacatggaaagacatccatacaaacac-3′; ROP18-D394A: 5′-gctcagggaattgtgcatacggctatcaaaccggcgaatttcctc-3′.

### Expression and Purification of Recombinant ROP18

RH-derived ROP18 was expressed as a N-terminal GST fusion protein from pGEX-4T-2 in *E. coli* BL21-CodonPlus (Stratagene) upon overnight induction with 0.1 mM IPTG at 18°C. The cells were lysed in PBS containing 2 mM DTT, 1% Triton X-114 and Complete Mini Protease Inhibitor Cocktail EDTA free (Roche) using a French press (Thermo Scientific). Cleared lysates were purified on a GSTrap FF glutathione Sepharose affinity column (GE Healthcare) in PBS containing 2 mM DTT and 1% Triton X-114. The GST-ROP18 fusion protein was eluted with PBS containing 2 mM DTT, 1% Triton X-114, and 10 mM reduced glutathione. Fractions containing ROP18 were subjected to size exclusion chromatography (Superdex 200; GE Healthcare) in PBS and 2 mM DTT. The remaining Triton X-114 was removed by phase separation at 37°C for 2 min and centrifugation, with ROP18 recovered from the aqueous phase. The protein was concentrated with a Vivaspin centrifugal concentrator (Sartorius). Amounts added to assays are cited as volume of the concentrate. The approximate concentration estimated in a Coomassie spot test [48] was 20 ng/µl.

### In Vitro Kinase Assay

Bacterially derived, purified GST-ROP18 was coincubated with wt or T102A, T108A, and T102/108A Irga6 mutants in 25 mM Tris/HCl (pH 7.5) and 15 mM MgCl_2_ for 30 min at 30°C. Each 50 µl reaction contained either 2 µCi of γ^32^P-ATP (Hartmann) for autoradiography or 1 mM unlabelled ATP for Western blot analysis. Samples were immediately boiled in sample buffer (80 mM Tris/HCl [pH 6.8], 5 mM EDTA, 4% SDS, 34% sucrose, 40 mM DTT, 0.002% bromophenol blue) for 5 min at 95°C and proteins were resolved by SDS-PAGE. Gels were dried and exposed to Biomax MS film (Kodak) or subjected to Western blot analysis.

### Yeast Two-Hybrid Assay

The yeast two-hybrid assay was performed as described previously [16].

### Metabolic Labelling, Immunoprecipitation, and Autoradiography

Cells were seeded as above onto 6 cm dishes, induced with IFNγ, washed, and preincubated in phosphate- or methionine/cysteine-free medium for 0.5 h before labelling with 100 µCi/ml ^33^P- or ^32^P-phosphoric acid, ^35^S-methionine/cysteine (all Hartmann) or 200 µCi/ml ^3^H-myristic acid (Perkin Elmer). After 1 h cells were infected, in the continued presence of label, with the indicated *T. gondii* strains. After infection for 2 h, cells were washed twice with ice-cold PBS and lysed in 500 µl 0.5% NP-40, 140 mM NaCl, 2 mM Tris/HCl (pH 7.6) containing protease and phosphatase inhibitors (Complete, Mini, EDTA free, and PhosSTOP, Roche) for 30 min on ice. Postnuclear lysates were incubated in the presence of specific antibodies or antisera overnight followed by 2 h incubation with 50 µl of protein A–Sepharose (Amersham) both at 4°C. Beads were washed once with 150 mM NaCl, 10 mM Tris/HCl (pH 7.6) and once with 10 mM Tris/HCl (pH 8) and either stored at −80°C or immediately boiled in sample buffer (80 mM Tris/HCl [pH 6.8], 5 mM EDTA, 4% SDS, 34% sucrose, 40 mM DTT, 0.002% bromophenol blue) for 5 min at 95°C. Proteins were resolved by SDS-PAGE, and gels were dried and exposed to Biomax MS film (Kodak) or imaged using a FLA3000 phosphorimager (Amersham). For analysis of ^3^H-labelled proteins the gel was preincubated with Autoradiography Enhancer (Perkin Elmer) for 1 h at room temperature and intensively washed before autoradiography of the dried gel.

### Immunoreagents

Rabbit antisera were raised at Innovagen AB against the following Irga6 phosphopeptides: Irga6 amino acids 97–107 (NH_2_-) C-EGAAK(pT102)GVVEV (-CONH_2_), (serum 87555), and Irga6 amino acids 103–113 (NH_2_-) C-GVVEV(pT108)MERHP (-CONH_2_) (serum 87558). In both cases, the N-terminal cysteine was added for conjugation to carrier (KLH). The 5th bleeds of each antiserum were first absorbed on the unphosphorylated peptide, and then affinity-purified on the phosphopeptide. Other immunoreagents used were: 10D7 and 10E7 mouse monoclonal antibodies [24] and 165 rabbit antiserum [23] against Irga6, anti-Irgb6 mouse monoclonal B34 [49], anti-Irgb10 rabbit serum 939/4 raised against recombinant full-length Irgb10, anti-ctag1 2600 rabbit antiserum [11], and anti-calnexin antiserum (Calbiochem). Second-stage antibodies were: Alexa 488 and Alexa 555 labelled donkey anti-mouse and anti-rabbit sera (Molecular Probes), goat anti-mouse-HRP (Pierce), and donkey anti-rabbit-HRP (GE Healthcare) antibodies.

### Immunofluorescence Microscopy and Analysis

For immunofluorescence microscopy, uninduced or IFNγ-induced cells grown on coverslips were infected or not with *T. gondii*, washed in PBS, fixed in PBS/3% paraformaldehyde for 15 min at room temperature and permeabilised in ice-cold methanol or 0.1% saponin at room temperature for 20 min before immunostaining. Vacuoles containing intracellular tachyzoites were identified from the characteristic phase contrast image. Microscopy and image analysis were performed essentially according to Hunn et al. [16]. All slides were counted double-blind, some independently by two observers. 100–500 vacuoles were counted in two to four independent experiments. This precaution is an essential control on observer bias in all nonautomated assessment of fluorescent images. Individual vacuoles for scoring were identified by a systematic scanning procedure in phase contrast and scored positive or negative for specific immunoreagents. Photographs of 20–40 vacuoles were analysed for fluorescence intensity according to Khaminets et al. [12]. Exposure times are indicated in brackets.

### Statistical Analyses

Statistical analyses were performed as described previously [12].

### Oligomerisation Assay

Oligomerisation of Irga6 was monitored by conventional light scattering as described earlier [16].

### Nucleotide Hydrolysis Assay

The nucleotide hydrolysis was measured by thin layer chromatography and autoradiography as described earlier [16].

### Nucleotide Binding Measurement

The nucleotide binding affinities were determined by equilibrium titration with mant-labelled nucleotides as described earlier [16].

### Mass Spectrometric Identification of Phosphorylated Peptides

Irga6 proteins immunoprecipitated from IFNγ-stimulated and *T. gondii* RH-YFP or RH infected cells were separated by SDS-PAGE and subsequently stained overnight in colloidal Coomassie blue solution. After destaining the gel in H_2_O, phosphorylated and nonmodified Irga6 bands were cut out and immediately subjected to MALDI and Nano-LC ESI- MS/MS mass spectrometry.

### Tryptic In-Gel Digest

SDS-PAGE bands of interest were digested as described elsewhere [50]. In brief, bands of interest were cut out and minced. After destaining with 50% 10 mM NH_4_HCO_3_/50% acetonitrile (ACN) at 55°C and dehydration in 100%ACN, gel pieces were equilibrated with 10 mM NH_4_HCO_3_ containing porcine trypsin (12.5 ng/µl; Promega, Mannheim) on ice for 2 h. Excess trypsin solution was removed and hydrolysis was performed for 4 h at 37°C in 10 mM NH_4_HCO_3_. Digests were acidified with 5% trifluoroacetic acid (TFA) and the gel pieces were extracted twice with 0.1% TFA and then with 60% ACN/40% H_2_O/0.1% TFA followed by a two-step treatment using 100% ACN. Extractions were combined, concentrated by vacuum centrifugation, and desalted according to Rappsilber et al. [51].

### Nano-LC ESI-MS/MS

Experiments were performed with an LTQ Orbitrap Discovery mass spectrometer (Thermo) coupled to a split-less Eksigent nano-LC system. Intact peptides were detected in the Orbitrap at 30,000 resolution in the mass-to-charge (m/z) range 400–2000 using m/z 445.120025 as a lock mass. Up to five CID spectra were acquired following each full scan. Peptides were separated on a 10 cm, 75 µm C18 reversed phase column (Proxeon) within 140 min at a flow rate of 200 nl/min (buffer A: 0.5% acetic acid, buffer B: 0.5% acetic acid, 80% ACN).

### MALDI Mass Spectrometry

For peptide mass fingerprinting, 2500 spectra in the mass-to-charge (m/z) range of 700–4,000 were acquired on a 4800 Plus MALDI-TOF/TOF Analyzer (Applied Biosystems) in the reflector positive mode using α-cyano-4-hydroxycinnamic acid (CHCA) (10 mg/ml, 50% ACN in 0.1% aqueous TFA). Automatic annotation of monoisotopic peptide signals in tryptic digests was performed using internal calibration on trypsin autolysis peaks at m/z 842 and 2211.

### Peptide and Protein Identification

Mascot 2.2 (Matrix Science) was used for protein identification by searching the Swissprot database of *Mus musculus* [52]. Mass tolerance for intact peptide masses was 20 ppm for MALDI MS data and 10 ppm for Orbitrap data, respectively. Mass tolerance for fragment ions detected in the linear ion trap was 0.8 Da. Oxidation of methionine and phosphorylation of serine, threonine, and tyrosine were set as variable modifications.

### Software

Swiss-PDBViewer [53] and PyMOL v0.99 (DeLano Scientific) were used for generation of crystal structure images. The multisequence alignment was generated with ClustalW via the EBI server (http://www.ebi.ac.uk/Tools/msa/clustalw2/) using the default settings and edited and shaded with GeneDoc [54]. SigmaPlot v9 (Systat) was used for dissociation constant calculation. AIDA Image Analyser v3 (Raytest) and ImageQuant TL v7 (GE Healthcare) were used for quantification of photostimulated luminescence. MS spectra analysis and peak annotation was performed using the MaxQuant software version 1.1.1.2.

## Supporting Information

Figure S1 **Irga6-T102 and -T108 phosphopeptides identified by mass spectrometry.** (A) Reversed phase chromatogram of the peptides with m/z 608.767 (mass tolerance 5 ppm) shown in Figure 2A, derived from immunoprecipitated, phosphorylated Irga6 after SDS-PAGE separation, tryptic digest and nano-LC ESI-MS/MS acquired on a LTQ/Orbitrap mass spectrometer. This m/z corresponds to two monophosphorylated peptides with the sequence TGVVEVTMER that eluted as two distinct peaks. (B and C) CID fragment spectrum of two peptides with m/z 608.767 separated in (A). For the first elution peak (Mascot ion score 31, delta mass +0.21 ppm) the masses of the fragment ions y4–y9 that contain T108 indicate that this residue is phosphorylated whereas the fragment ions b3–b5 containing T102 show no modification (B). For the second elution peak (Mascot ion score 37, delta mass −0.7 ppm) the masses of the fragment ions b3–b6 containing T102 indicate that this residue is phosphorylated whereas the fragment ions y5–y7 and y9 contain T108 and show no modification (C). Peaks labelled with an asterisk (*) correspond to fragment ions displaying a neutral loss of phosphoric acid (H_3_PO_4_, 98 Da).
(1.02 MB TIF)

Figure S2 **Specificity of rabbit anti-phosphothreonine antibodies anti-pT102 and anti-pT108 demonstrated with** ***Irga6*** **-deficient cells.** (A) Anti-pT102 and anti-pT108 antibodies detect phosphorylated Irga6 (black arrowhead) in Western blot only from IFNγ-induced wt MEFs infected with virulent RH-YFP strain *T. gondii*. No signals were detected in RH-YFP-infected, IFNγ-induced MEFs from *Irga6*-deficient (*Irga6* ^−/−^) mice. (B) Anti-pT102 and anti-pT108 antibodies detect phosphorylated Irga6 at the PV in IFNγ-induced wt MEFs infected with virulent RH strain *T. gondii*. Immunofluorescent signal intensities were measured on 20 vacuoles. Negligible signals were detected at the vacuole in IFNγ-induced, RH-infected, *Irga6*-deficient MEFs.
(0.59 MB TIF)

Figure S3 **Specificity control of rabbit anti-phosphothreonine antibodies anti-pT102 and anti-pT108 for** **Figure 6** **.** Rabbit anti-phosphothreonine antibodies give no immunofluorescent signals on parasitophorous vacuoles of virulent (RH-YFP, upper two rows) or avirulent (ME49, lower two rows) infected MEFs in the absence of induction by IFNγ. Intracellular *T. gondii* are indicated by arrows and individual exposure times are indicated in brackets.
(2.27 MB TIF)

Figure S4 **Specificity control of rabbit anti-phosphothreonine antibodies anti-pT102 and anti-pT108 for** **Figure 8** **.** Rabbit anti-phosphothreonine antibodies give no immunofluorescent signals on parasitophorous vacuoles of CTG strains transgenic for active virulent ROP18 (CTG V1), kinase-dead ROP18-D394A (CTG L1) or empty vector (CTG Ble) in the absence of induction by IFNγ. Intracellular *T. gondii* are indicated by arrows and individual exposure times are indicated in brackets.
(2.10 MB TIF)

Figure S5 **Direct interaction of ROP18 with Irga6.** Yeast two-hybrid analysis of the interaction of wt RH strain ROP18 and kinase-dead ROP18-D394A with Irga6. Growth on selective medium (SD medium lacking leucine, tryptophan, and histidine containing 1 mM 3-amino-1,2,4-triazole) indicative of protein–protein interaction is shown (day 20). Irga6 self-interaction [16] was used as a positive control and the empty expression vectors as negative controls.
(1.17 MB TIF)

Figure S6 **Irgb6 and Irgb10 are phosphorylated upon type I virulent strain infection.** Irgb6 and Irgb10 were immunoprecipitated from IFNγ-induced or transiently transfected, metabolically labelled L929 cells and infected with virulent *T. gondii* strain RH-YFP or CTG transgenic *T. gondii* strains. (A) A weak ^32^P-inorganic phosphate labelled band corresponding to Irgb6 (black arrowhead on the left) was immunoprecipitated with mouse monoclonal anti-Irgb6 antibody, B34, only from IFNγ-induced cells. A very weakly labelled nonspecific band running above the positive control Irga6 (black arrowhead on the right) protein (precipitated with 10E7 antibody) is indicated by an asterisk. (B) A montage of autoradiograms showing immunoprecipitation of Irga6 (left panel), Irgb10 (middle panel) from RH-YFP-infected, CTG transgenic strain-infected, or uninfected L929 cells labelled with ^33^P-phosphoric acid. The Irga6 results serve as positive controls (black arrowhead on the left). Phosphorylated Irgb10 (black arrowhead on the left) could be reproducibly immunoprecipitated from uninfected, IFNγ-induced cells, but the signal was enhanced by RH-YFP infection. Two nonspecific labelled bands precipitated by the anti-Irgb10 antiserum are indicated with asterisks. The right panel shows the specificity of the rabbit anti-Irgb10 antiserum, precipitating Irgb10 but not Irga6 from ^35^S-methionine/cysteine metabolically labelled cells (open arrowheads on the right). Two nonspecifically immunoprecipitated labelled bands are indicated with asterisks. (C) Recombinant Irga6-ctag1 and Irgb10-ctag1 proteins were immunoprecipitated from transiently transfected, IFNγ-induced, ^33^P-labelled cells. Phosphorylation of Irgb10 could be demonstrated in uninfected cells but the signal intensity was strongly increased upon infection with virulent RH-YFP *T. gondii* (black arrowhead on the right). Phosphorylation of Irga6 was strictly dependent on infection and served as a positive control (black arrowhead on the left).
(1.55 MB TIF)

Table S1 **Nucleotide binding affinities of Irga6-T102 and -T108 mutants.** Dissociation constant (*K*_d_) measured by equilibrium titration with mant-nucleotides. The mean values and the standard deviation of at least two independent experiments are given.
(0.19 MB TIF)

## Abbreviations

CID: collision-induced dissociation
GBP: guanylate binding protein
IRG: immunity-related GTPase
MEF: mouse embryonic fibroblast
MS: mass spectrometry
PVM: parasitophorous vacuole membrane
wt: wild type
