# Correction: Phosphorylation of Mouse Immunity-Related GTPase (IRG) Resistance Proteins Is an Evasion Strategy for Virulent *Toxoplasma gondii*

**DOI:** 10.1371/journal.pbio.1002199

**Published:** 2015-07-09

**Authors:** Tobias Steinfeldt, Stephanie Könen-Waisman, Lan Tong, Nikolaus Pawlowski, Tobias Lamkemeyer, L. David Sibley, Julia P. Hunn, Jonathan C. Howard

The current published Figure S6C contains lanes from a single gel run that have been rearranged and spliced for the purposes of figure composition. The authors wish to correct Figure S6 to adequately show these splices by the addition of solid splice lines, and by correcting the legend for further clarity. The method of this figure composition does not affect the scientific understanding of the article. A corrected version of this figure, named S6 Fig, in addition to a corrected legend, are provided here.

## Supporting Information

S6 Fig Irgb6 and Irgb10 are phosphorylated upon type I virulent strain infection. Irgb6 and Irgb10 were immunoprecipitated from IFNγ-induced or transiently transfected, metabolically labelled L929 cells and infected with virulent *T*. *gondii* strain RH-YFP or CTG transgenic *T*. *gondii* strains. (A) A weak ^32^P-inorganic phosphate labelled band corresponding to Irgb6 (black arrowhead on the left) was immunoprecipitated with mouse monoclonal anti-Irgb6 antibody, B34, only from IFNγ-induced cells. A very weakly labelled nonspecific band running above the positive control Irga6 (black arrowhead on the right) protein (precipitated with 10E7 antibody) is indicated by an asterisk. (B) A montage of autoradiograms showing immunoprecipitation of Irga6 (left panel), Irgb10 (middle panel) from RH-YFP-infected, CTG transgenic strain-infected, or uninfected L929 cells labelled with ^33^P-phosphoric acid. The Irga6 results serve as positive controls (black arrowhead on the left). Phosphorylated Irgb10 (black arrowhead on the left) could be reproducibly immunoprecipitated from uninfected, IFNγ-induced cells, but the signal was enhanced by RH-YFP infection. Two nonspecific labelled bands precipitated by the anti-Irgb10 antiserum are indicated with asterisks. The right panel shows the specificity of the rabbit anti-Irgb10 antiserum, precipitating Irgb10 but not Irga6 from ^35^S-methionine/cysteine metabolically labelled cells (open arrowheads on the right). Two nonspecifically immunoprecipitated labelled bands are indicated with asterisks. (C) Recombinant Irga6-ctag1 and Irgb10-ctag1 proteins were immunoprecipitated from transiently transfected, IFNγ-induced, ^33^P-labelled cells. Phosphorylation of Irgb10 could be demonstrated in uninfected cells but the signal intensity was strongly increased upon infection with virulent RH-YFP *T*. *gondii* (black arrowhead on the right). Phosphorylation of Irga6 was strictly dependent on infection and served as a positive control (black arrowhead on the left). The panel is a montage of 4 lanes from a single autoradiogram, joined as indicated by vertical black lines.
(PDF)
