# Supplementary figures and images for: Phosphorylation of Mouse Immunity-Related GTPase (IRG) Resistance Proteins Is an Evasion Strategy for Virulent Toxoplasma gondii
Source: PLoS Biol. 2010 Dec 21;8(12):e1000576. doi: 10.1371/journal.pbio.1000576 (PMC3006384; doi:10.1371/journal.pbio.1000576)

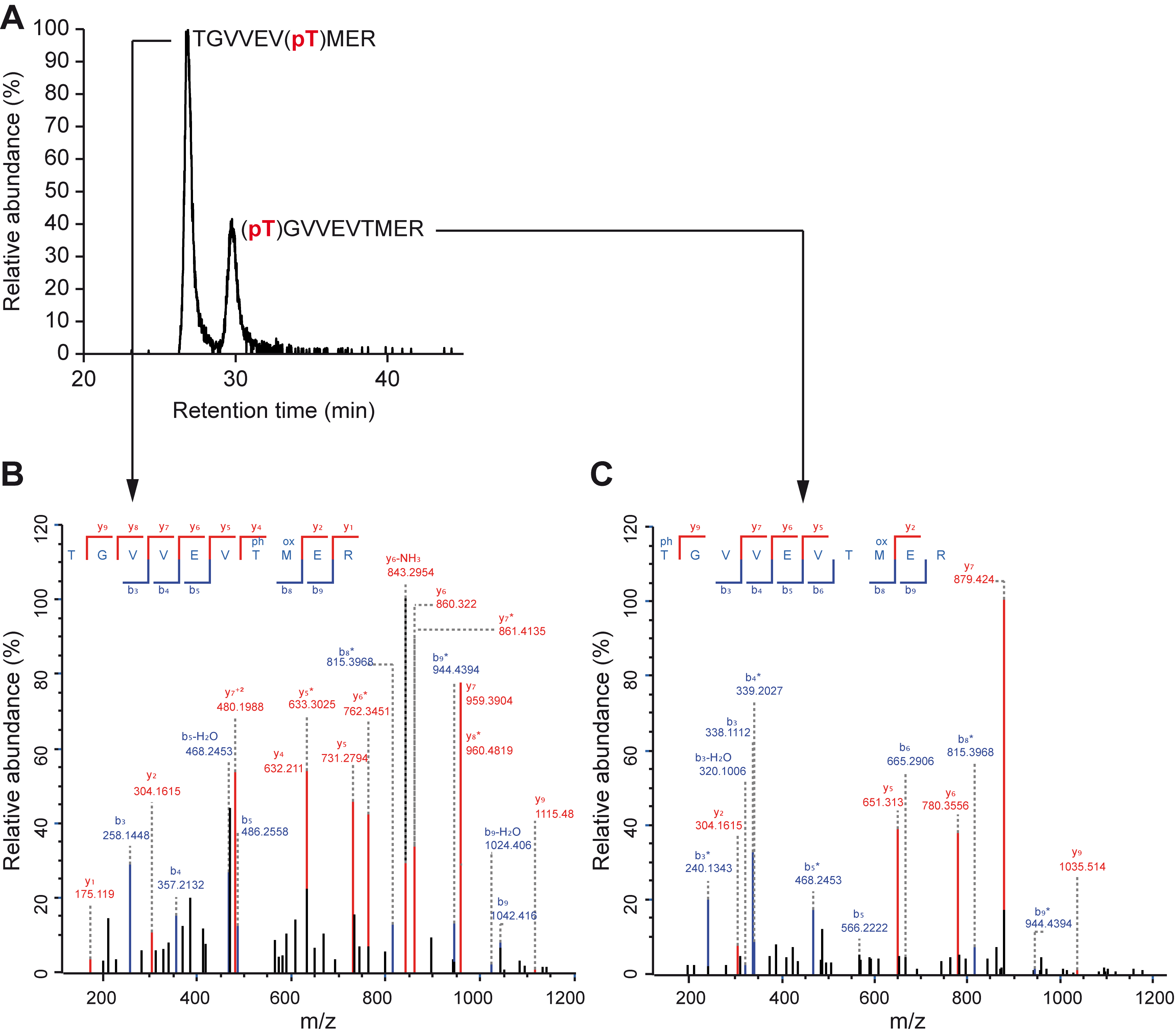

Supplement: Figure S1 — Irga6-T102 and -T108 phosphopeptides identified by mass spectrometry. (A) Reversed phase chromatogram of the peptides with m/z 608.767 (mass tolerance 5 ppm) shown in Figure 2A, derived from immunoprecipitated, phosphorylated Irga6 after SDS-PAGE separation, tryptic digest and nano-LC ESI-MS/MS acquired on a LTQ/Orbitrap mass spectrometer. This m/z corresponds to two monophosphorylated peptides with the sequence TGVVEVTMER that eluted as two distinct peaks. (B and C) CID fragment spectrum of two peptides with m/z 608.767 separated in (A). For the first elution peak (Mascot ion score 31, delta mass +0.21 ppm) the masses of the fragment ions y4–y9 that contain T108 indicate that this residue is phosphorylated whereas the fragment ions b3–b5 containing T102 show no modification (B). For the second elution peak (Mascot ion score 37, delta mass −0.7 ppm) the masses of the fragment ions b3–b6 containing T102 indicate that this residue is phosphorylated whereas the fragment ions y5–y7 and y9 contain T108 and show no modification (C). Peaks labelled with an asterisk (*) correspond to fragment ions displaying a neutral loss of phosphoric acid (H3PO4, 98 Da). (1.02 MB TIF) [file pbio.1000576.s001.tif]

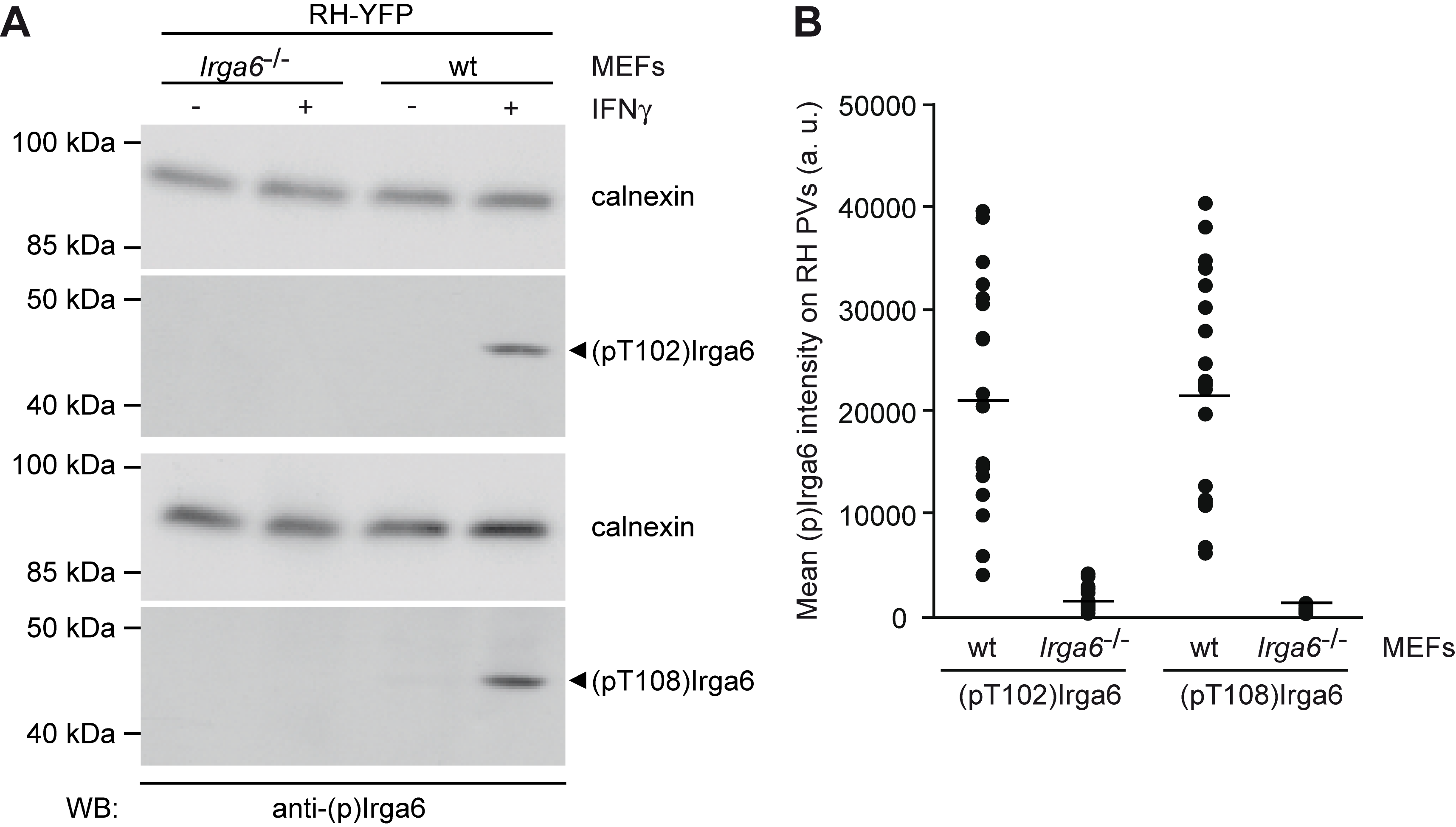

Supplement: Figure S2 — Specificity of rabbit anti-phosphothreonine antibodies anti-pT102 and anti-pT108 demonstrated with Irga6 -deficient cells. (A) Anti-pT102 and anti-pT108 antibodies detect phosphorylated Irga6 (black arrowhead) in Western blot only from IFNγ-induced wt MEFs infected with virulent RH-YFP strain T. gondii. No signals were detected in RH-YFP-infected, IFNγ-induced MEFs from Irga6-deficient (Irga6 −/−) mice. (B) Anti-pT102 and anti-pT108 antibodies detect phosphorylated Irga6 at the PV in IFNγ-induced wt MEFs infected with virulent RH strain T. gondii. Immunofluorescent signal intensities were measured on 20 vacuoles. Negligible signals were detected at the vacuole in IFNγ-induced, RH-infected, Irga6-deficient MEFs. (0.59 MB TIF) [file pbio.1000576.s002.tif]

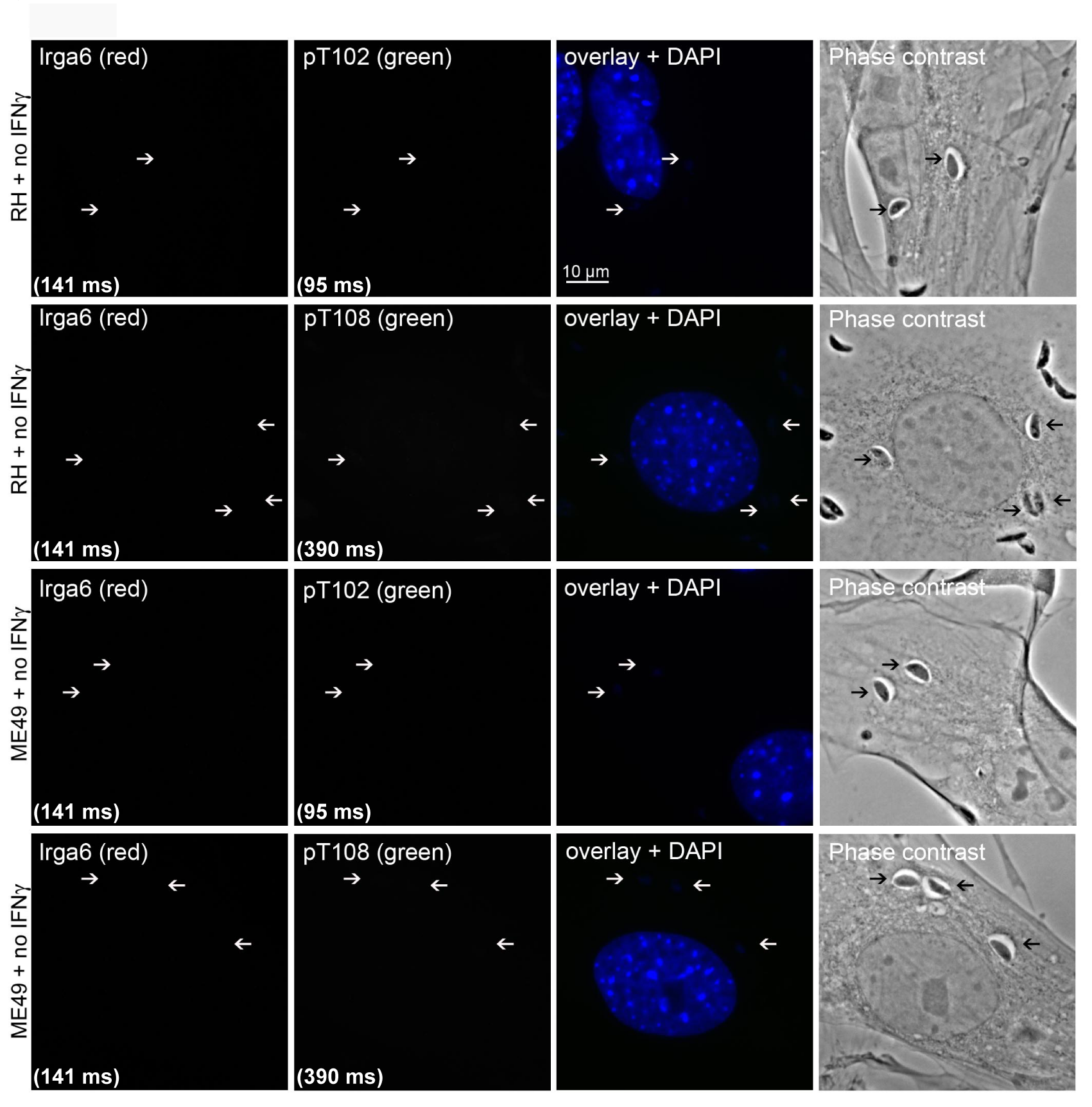

Supplement: Figure S3 — Specificity control of rabbit anti-phosphothreonine antibodies anti-pT102 and anti-pT108 for Figure 6 . Rabbit anti-phosphothreonine antibodies give no immunofluorescent signals on parasitophorous vacuoles of virulent (RH-YFP, upper two rows) or avirulent (ME49, lower two rows) infected MEFs in the absence of induction by IFNγ. Intracellular T. gondii are indicated by arrows and individual exposure times are indicated in brackets. (2.27 MB TIF) [file pbio.1000576.s003.tif]

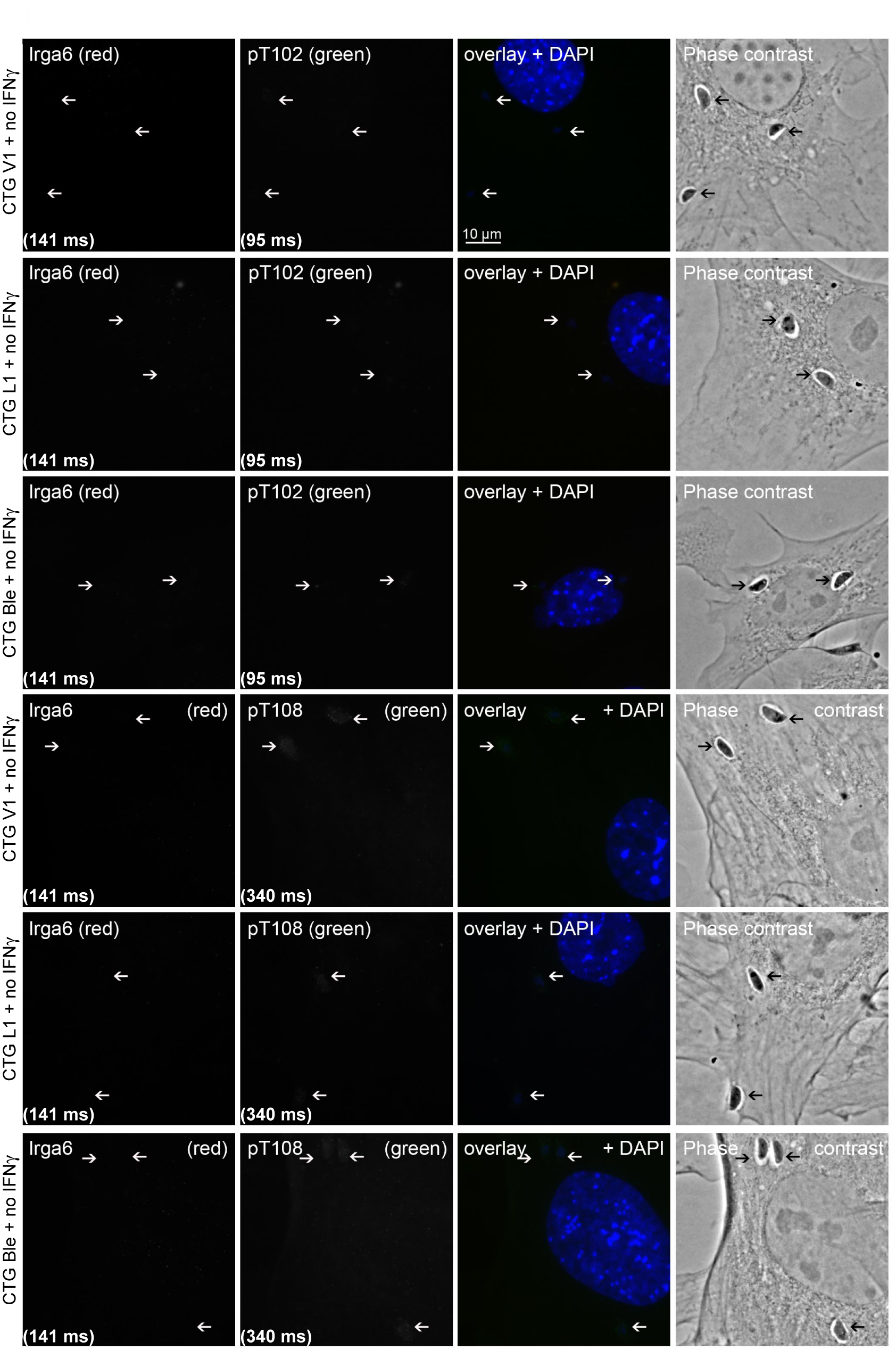

Supplement: Figure S4 — Specificity control of rabbit anti-phosphothreonine antibodies anti-pT102 and anti-pT108 for Figure 8 . Rabbit anti-phosphothreonine antibodies give no immunofluorescent signals on parasitophorous vacuoles of CTG strains transgenic for active virulent ROP18 (CTG V1), kinase-dead ROP18-D394A (CTG L1) or empty vector (CTG Ble) in the absence of induction by IFNγ. Intracellular T. gondii are indicated by arrows and individual exposure times are indicated in brackets. (2.10 MB TIF) [file pbio.1000576.s004.tif]

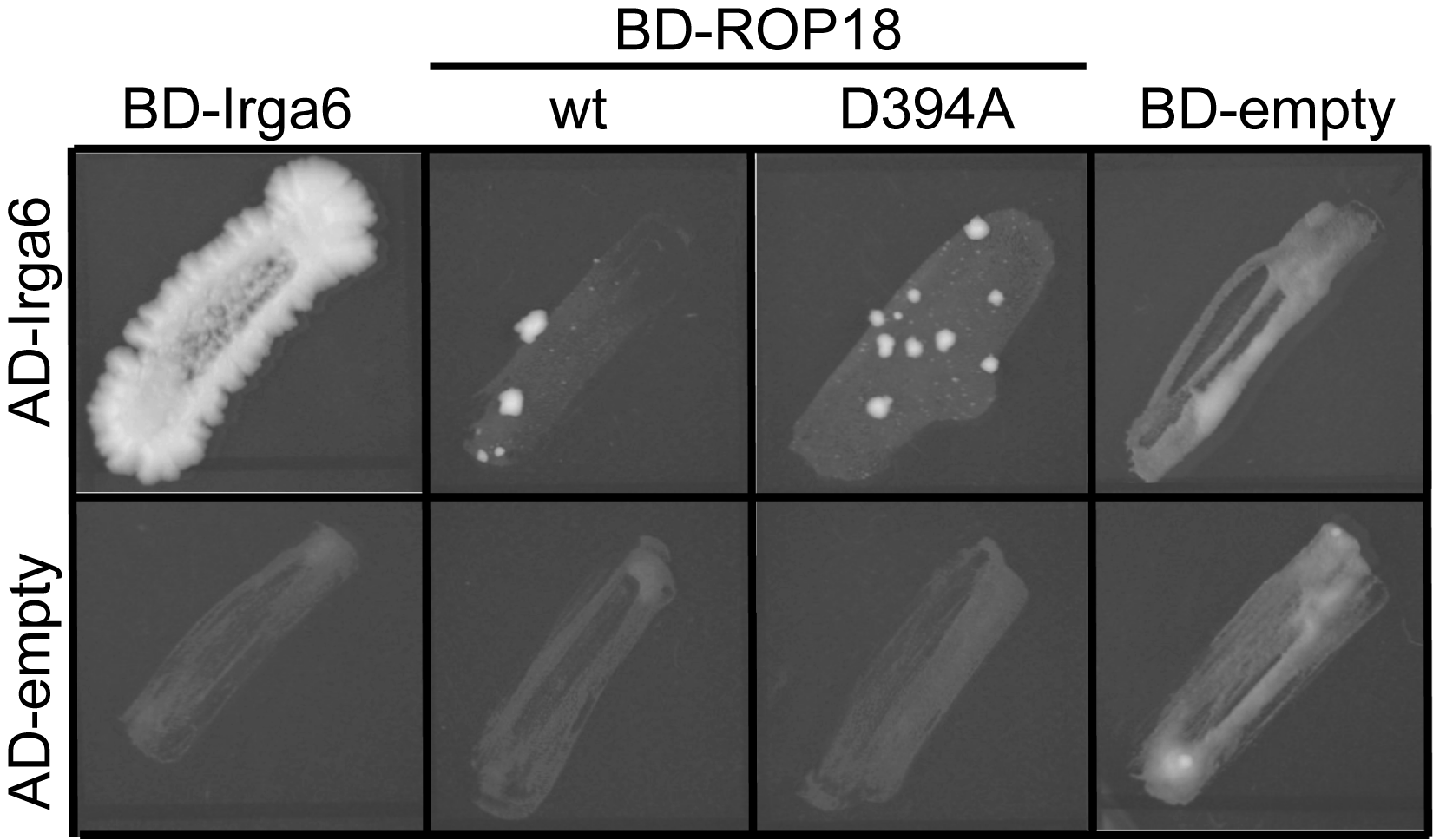

Supplement: Figure S5 — Direct interaction of ROP18 with Irga6. Yeast two-hybrid analysis of the interaction of wt RH strain ROP18 and kinase-dead ROP18-D394A with Irga6. Growth on selective medium (SD medium lacking leucine, tryptophan, and histidine containing 1 mM 3-amino-1,2,4-triazole) indicative of protein–protein interaction is shown (day 20). Irga6 self-interaction [16] was used as a positive control and the empty expression vectors as negative controls. (1.17 MB TIF) [file pbio.1000576.s005.tif]

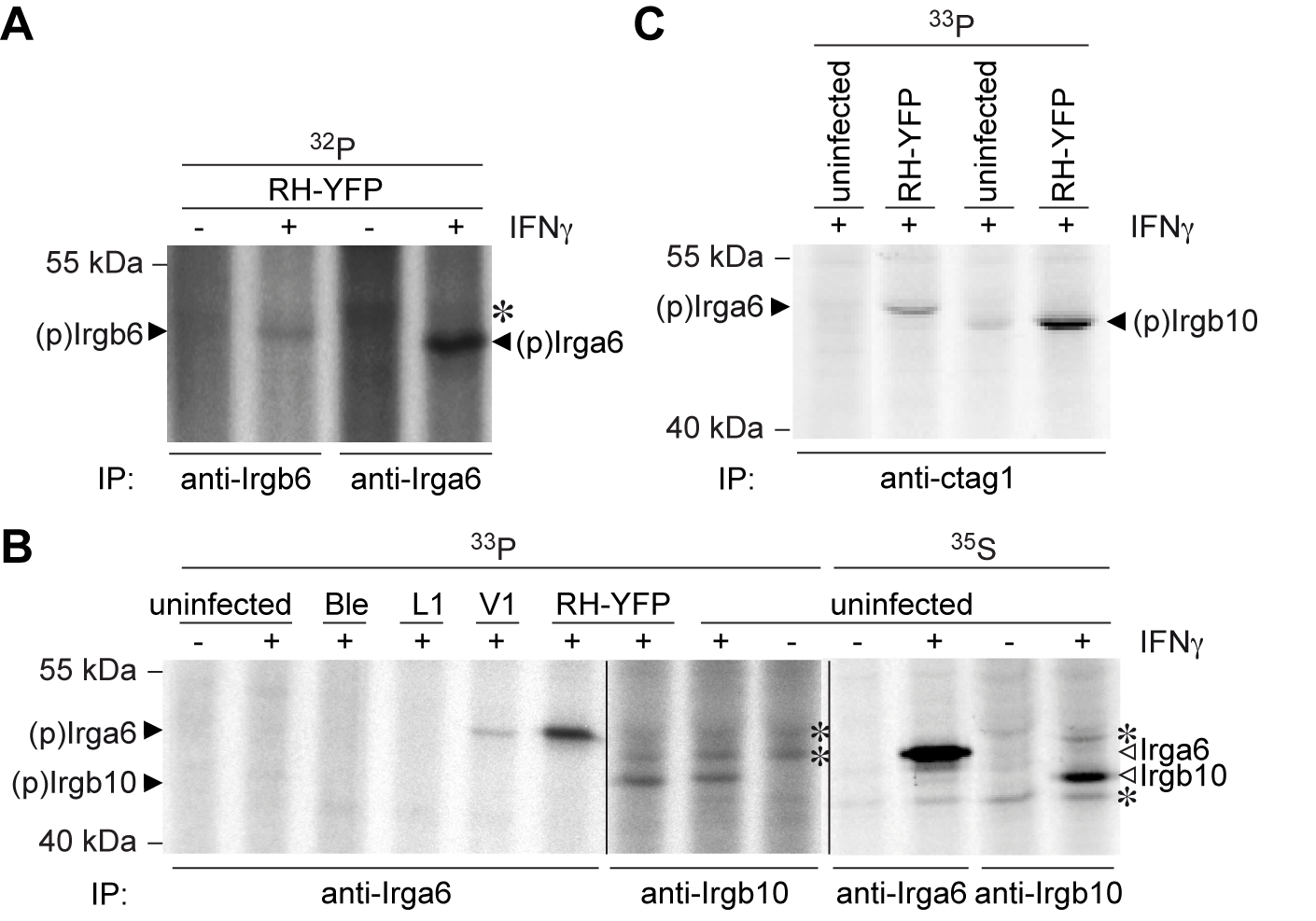

Supplement: Figure S6 — Irgb6 and Irgb10 are phosphorylated upon type I virulent strain infection. Irgb6 and Irgb10 were immunoprecipitated from IFNγ-induced or transiently transfected, metabolically labelled L929 cells and infected with virulent T. gondii strain RH-YFP or CTG transgenic T. gondii strains. (A) A weak 32P-inorganic phosphate labelled band corresponding to Irgb6 (black arrowhead on the left) was immunoprecipitated with mouse monoclonal anti-Irgb6 antibody, B34, only from IFNγ-induced cells. A very weakly labelled nonspecific band running above the positive control Irga6 (black arrowhead on the right) protein (precipitated with 10E7 antibody) is indicated by an asterisk. (B) A montage of autoradiograms showing immunoprecipitation of Irga6 (left panel), Irgb10 (middle panel) from RH-YFP-infected, CTG transgenic strain-infected, or uninfected L929 cells labelled with 33P-phosphoric acid. The Irga6 results serve as positive controls (black arrowhead on the left). Phosphorylated Irgb10 (black arrowhead on the left) could be reproducibly immunoprecipitated from uninfected, IFNγ-induced cells, but the signal was enhanced by RH-YFP infection. Two nonspecific labelled bands precipitated by the anti-Irgb10 antiserum are indicated with asterisks. The right panel shows the specificity of the rabbit anti-Irgb10 antiserum, precipitating Irgb10 but not Irga6 from 35S-methionine/cysteine metabolically labelled cells (open arrowheads on the right). Two nonspecifically immunoprecipitated labelled bands are indicated with asterisks. (C) Recombinant Irga6-ctag1 and Irgb10-ctag1 proteins were immunoprecipitated from transiently transfected, IFNγ-induced, 33P-labelled cells. Phosphorylation of Irgb10 could be demonstrated in uninfected cells but the signal intensity was strongly increased upon infection with virulent RH-YFP T. gondii (black arrowhead on the right). Phosphorylation of Irga6 was strictly dependent on infection and served as a positive control (black arrowhead on the left). (1.55 MB [file pbio.1000576.s006.tif]

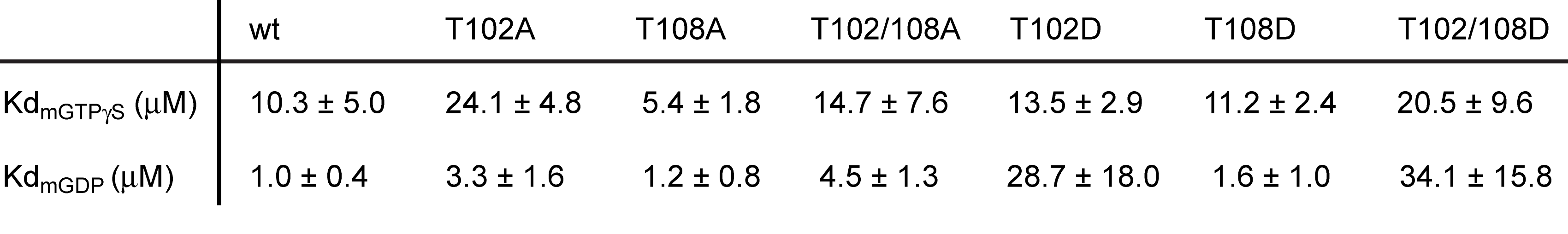

Supplement: Table S1 — Nucleotide binding affinities of Irga6-T102 and -T108 mutants. Dissociation constant (K d) measured by equilibrium titration with mant-nucleotides. The mean values and the standard deviation of at least two independent experiments are given. (0.19 MB TIF) [file pbio.1000576.s007.tif]
