# Supplementary figures and images for: Correction: Phosphorylation of Mouse Immunity-Related GTPase (IRG) Resistance Proteins Is an Evasion Strategy for Virulent Toxoplasma gondii
Source: PLoS Biol. 2015 Jul 9;13(7):e1002199. doi: 10.1371/journal.pbio.1002199 (PMC4497683; doi:10.1371/journal.pbio.1002199)

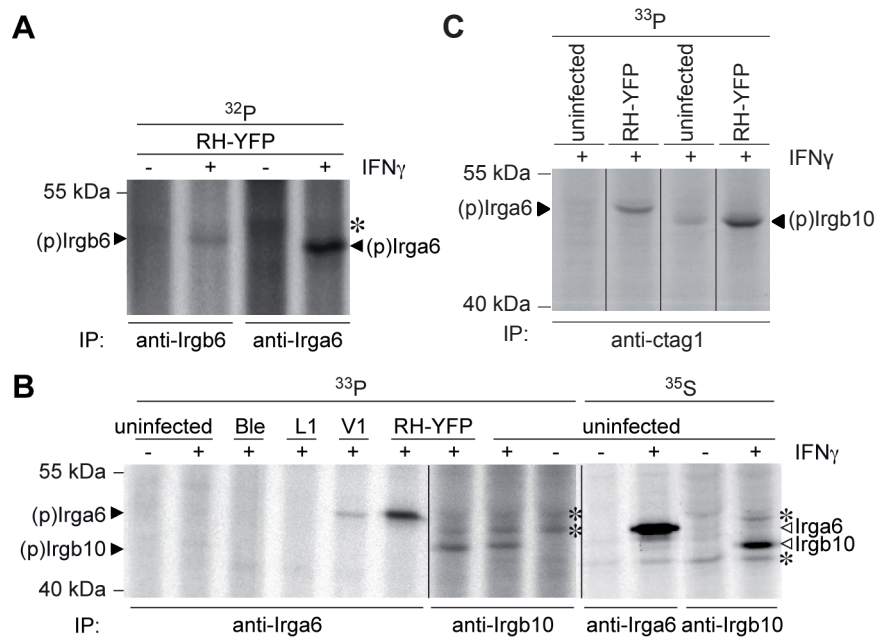

Supplement: S6 Fig — Irgb6 and Irgb10 were immunoprecipitated from IFNγ-induced or transiently transfected, metabolically labelled L929 cells and infected with virulent T. gondii strain RH-YFP or CTG transgenic T. gondii strains. (A) A weak 32P-inorganic phosphate labelled band corresponding to Irgb6 (black arrowhead on the left) was immunoprecipitated with mouse monoclonal anti-Irgb6 antibody, B34, only from IFNγ-induced cells. A very weakly labelled nonspecific band running above the positive control Irga6 (black arrowhead on the right) protein (precipitated with 10E7 antibody) is indicated by an asterisk. (B) A montage of autoradiograms showing immunoprecipitation of Irga6 (left panel), Irgb10 (middle panel) from RH-YFP-infected, CTG transgenic strain-infected, or uninfected L929 cells labelled with 33P-phosphoric acid. The Irga6 results serve as positive controls (black arrowhead on the left). Phosphorylated Irgb10 (black arrowhead on the left) could be reproducibly immunoprecipitated from uninfected, IFNγ-induced cells, but the signal was enhanced by RH-YFP infection. Two nonspecific labelled bands precipitated by the anti-Irgb10 antiserum are indicated with asterisks. The right panel shows the specificity of the rabbit anti-Irgb10 antiserum, precipitating Irgb10 but not Irga6 from 35S-methionine/cysteine metabolically labelled cells (open arrowheads on the right). Two nonspecifically immunoprecipitated labelled bands are indicated with asterisks. (C) Recombinant Irga6-ctag1 and Irgb10-ctag1 proteins were immunoprecipitated from transiently transfected, IFNγ-induced, 33P-labelled cells. Phosphorylation of Irgb10 could be demonstrated in uninfected cells but the signal intensity was strongly increased upon infection with virulent RH-YFP T. gondii (black arrowhead on the right). Phosphorylation of Irga6 was strictly dependent on infection and served as a positive control (black arrowhead on the left). The panel is a montage of 4 lanes from a single autoradiogram, joined as indicated [file pbio.1002199.s001.pdf]
